# Global MYCN Transcription Factor Binding Analysis in Neuroblastoma Reveals Association with Distinct E-Box Motifs and Regions of DNA Hypermethylation

**DOI:** 10.1371/journal.pone.0008154

**Published:** 2009-12-04

**Authors:** Derek M. Murphy, Patrick G. Buckley, Kenneth Bryan, Sudipto Das, Leah Alcock, Niamh H. Foley, Suzanne Prenter, Isabella Bray, Karen M. Watters, Desmond Higgins, Raymond L. Stallings

**Affiliations:** 1 Department of Cancer Genetics, Royal College of Surgeons in Ireland, Dublin, Ireland; 2 Children's Research Centre, Our Lady's Children's Hospital, Dublin, Ireland; 3 Conway Institute of Biomolecular and Biomedical Research, University College Dublin, Dublin, Ireland; City of Hope National Medical Center, United States of America

## Abstract

**Background:**

Neuroblastoma, a cancer derived from precursor cells of the sympathetic nervous system, is a major cause of childhood cancer related deaths. The single most important prognostic indicator of poor clinical outcome in this disease is genomic amplification of *MYCN*, a member of a family of oncogenic transcription factors.

**Methodology:**

We applied MYCN chromatin immunoprecipitation to microarrays (ChIP-chip) using *MYCN* amplified/non-amplified cell lines as well as a conditional knockdown cell line to determine the distribution of MYCN binding sites within all annotated promoter regions.

**Conclusion:**

Assessment of E-box usage within consistently positive MYCN binding sites revealed a predominance for the CATGTG motif (*p*<0.0016), with significant enrichment of additional motifs CATTTG, CATCTG, CAACTG in the *MYCN* amplified state. For cell lines over-expressing MYCN, gene ontology analysis revealed enrichment for the binding of MYCN at promoter regions of numerous molecular functional groups including DNA helicases and mRNA transcriptional regulation. In order to evaluate MYCN binding with respect to other genomic features, we determined the methylation status of all annotated CpG islands and promoter sequences using methylated DNA immunoprecipitation (MeDIP). The integration of MYCN ChIP-chip and MeDIP data revealed a highly significant positive correlation between MYCN binding and DNA hypermethylation. This association was also detected in regions of hemizygous loss, indicating that the observed association occurs on the same homologue. In summary, these findings suggest that MYCN binding occurs more commonly at CATGTG as opposed to the classic CACGTG E-box motif, and that disease associated over expression of MYCN leads to aberrant binding to additional weaker affinity E-box motifs in neuroblastoma. The co-localization of MYCN binding and DNA hypermethylation further supports the dual role of MYCN, namely that of a classical transcription factor affecting the activity of individual genes, and that of a mediator of global chromatin structure.

## Introduction

MYCN is one member of a family of oncogenic transcription factors that also include c-MYC and MYCL. These proteins bind DNA in a sequence specific manner in order to regulate normal growth and differentiation during development [1]. The *myc* gene family is only a subset of a much larger super family of genes that encodes DNA binding basic helix-loop-helix proteins (bHLH). Proteins containing the bHLH motif are known to be involved in a diverse range of cellular processes including proliferation, differentiation and morphogenesis. bHLH proteins can bind DNA as homodimers, but heterodimerization with other bHLH proteins has been shown to dramatically increase DNA binding efficiency [2].

High level genomic amplification of the *MYCN* gene occurs in approximately 20 to 25% of neuroblastoma (NB), a highly genetically heterogeneous childhood cancer derived from precursor cells of the sympathetic nervous system. *MYCN* amplification is the single most important prognostic indicator of poor clinical outcome [3]. Currently, patients with *MYCN* amplified neuroblastoma tumors have less than a 30% chance of 5-year survival, thus identification of downstream MYCN targets is critically important for the development of alternative treatment regimens and improving patient survival.

Analysis of gene expression in NB cell lines where MYCN levels can be experimentally manipulated have identified many genes and miRNA sequences whose expression is altered in response to changes in MYCN levels [4]–[6]. Distinguishing direct versus indirect effects based on expression profiling, however, is difficult since MYCN regulates other transcription factors as well as regulatory RNAs such as miRNAs. A number of studies have used techniques such as chromatin immunoprecipitation (ChIP) to experimentally confirm MYCN binding to the promoter regions of specific genes [7]–[9], and more recent studies have identified MYCN binding sites in proximity to miRNA promoter regions [10]. Analysis of the relationship between MYCN binding and expression of the target gene sequence, however, is not straightforward, as MYCN binding throughout the genome is far more ubiquitous than previously realized, with large numbers of intergenic binding sites indicating a more general role for MYCN in maintaining euchromatin structure that is independent of its role in regulating the expression of specific genes [11].

Here, we have performed MYCN ChIP-chip studies on NB cell lines using a set of microarrays containing all annotated human gene promoter regions, as well as a custom tiling array covering selected miRNA loci and intergenic regions. Assessment of E-box usage and gene ontology enrichment analysis was carried out on identified MYCN binding sites. Finally, using methylation dependent immunoprecipitation (MeDIP), we also determine the overall methylation status of MYCN binding sites and observed a striking correlation between MYCN binding and DNA hypermethylation status in the neuroblastoma cell lines studied.

## Results

To identify high confidence MYCN transcription factor binding sites within promoter sequences across the genome, we performed ChIP-chip using two antibodies that were reported in previous MYCN ChIP-chip or ChIP-Seq studies, namely NCMII-100 [11] and B84b [12], [13]. Given that these mouse monoclonal antibodies are raised against different epitopes of the MYCN protein, we reasoned that MYCN binding sites identified independently by both antibodies are more likely to be genuine. A pair-wise comparison of log_2_ ratios from ChIP-chip experiments using the NB cell line Kelly, revealed a good correlation across experiments (R = 0.8) between independent antibodies, confirming that this approach was able to reproducibly detect MYCN binding sites (Figure S1A and B ).

### Characterisation of MYCN Transcription Factor Binding Sites

**Figure 1:**
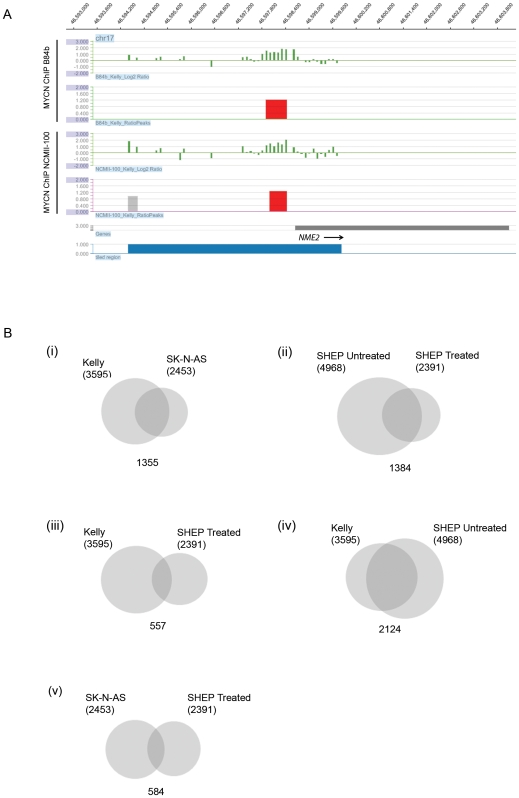
MYCN ChIP-chip. (A) Identification of MYCN binding upstream of *NME2*. The scale across the top of the panel indicates the base pair position on chromosome 17. Fluorescent intensity of probes from experiments using 2 independent MYCN antibodies around the *NME2* promoter are expressed as log_2_ ratios (green bars) and high confidence MYCN peaks (red bars) as identified by the NimbleScan peak finding algorithm. Position of the *NME2* transcript and the region tiled on the array are indicated by the bottom two panels. (B) Total number of MYCN peaks identified across neuroblastoma cell lines. Venn diagrams (i–v) display the number of MYCN peaks which is shared and unique to each cell line. Statistical significance of overlap for all comparisons was determined to be statistically significant (P<0.001) by Fisher's Exact Test.

ChIP-chip assays were performed on NB cell lines comprising a *MYCN* amplified (Kelly), non *MYCN* amplified (SK-N-AS) and a constitutively over-expressing MYCN cell line SHEP-21N which contains a *MYCN* trans-gene under the control of a tetracycline responsive repressor element [14] [referred hereafter as SHEP-untreated (high levels of MYCN) and SHEP-treated (low expression of MYCN), as illustrated in Figure S1 C and D]. A number of previously reported MYCN targets were positive for MYCN binding in our ChIP-chip experiments, confirming the validity of our two antibody approach, including *NME2* [13], [15], *CRABP-II* [16], *ZNF259* [11], *LIF* [17] and members of the mini-chromosome maintenance gene family [8]. Figure 1A illustrates MYCN binding to one of these previously published positive MYCN binding sites, *NME2.* Additional validation for the enrichment of MYCN in the antibody precipitated fraction was also obtained through qPCR based analysis of binding sites (Figure S2). The level of enrichment (∼2 to 4 fold) was similar to other transcription factors such as ZNF217 [18] and other MYCN semi-quantitative PCR validation experiments carried out by other groups [11], [13], consistent with the assertion that MYCN binds with a weak affinity to promoter sequences.

Figure 1B summarises the total number of MYCN peaks identified using the two independent MYCN antibodies and the proportion of shared peaks across the respective cell lines analysed using the NimbleGen 2-array promoter set. The total number of MYCN peaks identified in the various cell lines was generally consistent with the level of MYCN expression (Figure S1C–E). The *MYCN* amplified (MNA) cell line Kelly displayed 1,142 more positive MYCN peaks than the non-amplified cell line SK-N-AS, while sharing 38% of MYCN peaks [Fig. 1B(i)]. In addition, treating the SHEP cell line with doxycycline (dox) resulted in the reduction of MYCN peaks by 52% [Fig. 1B(ii)]. This reduction in the number of MYCN peaks is in contrast to a previous ChIP-chip study using a similar knockdown strategy for MYCN which observed a 99% decrease in the number of MYCN peaks [11]; however such differences in the decreased levels of MYCN binding may be due to experimental variations in the ChIP design, protocols and/or array designs used as well as the nature and extent of the loci studied.

**Figure 2:**
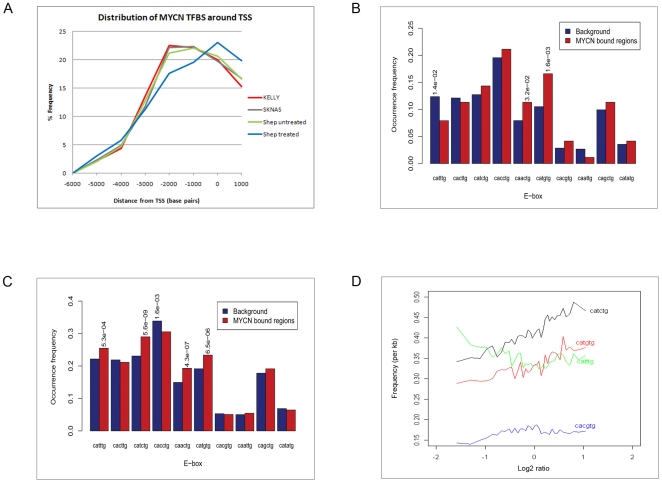
Distribution of MYCN transcription factor binding and E-box preference. (A) Percentage of MYCN binding sites around transcription start sites. X-axis displays the distance in base pairs from UCSC annotated transcriptional start sites. Y-axis represents the percentage frequency of positive MYCN binding sites identified in each neuroblastoma cell line. (B) Prevalence of E-box motifs within MYCN binding sites detected by ChIP-chip across all cell lines and both antibodies. Statistically significant bars are indicated with their respective *p*-values (C) Prevalence of E-box motifs within the MYCN binding sites detected by ChIP-chip in the *MYCN*-amplified state only. Statistically significant bars are indicated with their respective *p*-values. (D) Association of E-box frequency with raw fluorescent ratios in MYCN binding sites on the promoter array which were detected in the Kelly NB cell line. The number of E-boxes per kilobase (y-axis) is plotted against the raw array fluorescent ratio intensities.

Interestingly, the percentage overlap of positive MYCN peaks between Kelly and SHEP treated (20%) increased dramatically to 75% with MYCN over expression in SHEP untreated [Fig. 1B(iii & iv)], confirming that the over expression of MYCN in the SHEP-based system results in a binding pattern which has a high degree of similarity to that of a MNA cell line such as Kelly. In general, the low expressing MYCN cell lines (SK-N-AS & SHEP-treated) displayed a similar number of MYCN peaks, although when compared to each other only shared 25% of peaks [Fig. 1B(v)]. The overlap of MYCN binding sites between NB cell lines displayed in Figure 1B was found to be statistically significant (P<0.001). This was determined using the expected peak overlap frequency given the number of peaks retrieved in the individual ChIP-chip experiments and the number of regions on the microarray. Fisher's Exact Test was then used to calculate the P-value for each overlap based on the actual versus the expected frequencies. ChIP-chip reactions performed with Dyna-beads only and an isotype matched IgG mouse antibody using the 2-array promoter set and/or custom tiled arrays resulted in only random, minimal overlap with positive MYCN peaks (average overlap of 5.8%), indicating that the vast majority of the MYCN peaks were not artifactually generated. In order to study the distribution of MYCN binding around promoter sequences, identified peaks were plotted in relation to the distance from their annotated transcription start sites for the cell lines studied (Figure 2A). The distribution of MYCN sites was generally consistent across cell lines; however, upon treatment with dox the SHEP cell line displayed an inward shift towards the transcriptional start site, suggesting that both qualitative and quantitative changes in MYCN binding occur upon dox treatment. Results confirm that the majority of MYCN binding (average 80%) falls within −3kb to +1kb of the transcriptional start site, which is consistent with previous studies for MYCN and c-MYC [11], [19].

### E-box Usage in Neuroblastoma

E-box preference for putative MYCN binding within both the *MYCN* amplified and non-amplified NB cell lines was assessed by examining occurrences of all possible variants of the generic E-box motif, CA*NN*TG, within MYCN positive sites relative to the background sequence on the promoter microarrays. Significance was assessed using p-values derived from Fisher's Exact test. For this, we initially focussed on highly consistent MYCN binding sites common to all NB cell lines used in the study. A subset of 265 sites, which were below the selected false discovery rate (FDR) threshold in each cell line, were selected as the representative set of MYCN binding sites and subjected to further examination (Table S1). As illustrated in Figure 2B, the most significantly over-represented motifs were CATGTG (*p* = 0.0016) and CAACTG (*p* = 0.032), while the motif CATTTG (*p* = 0.014) was significantly under-represented. Next, we assessed if a shift occurred in E-box preference between the *MYCN*-amplified state, in which MYCN is highly expressed, and the non-amplified state, which would help explain the mechanism of oncogenesis induced by *MYCN* amplification. We examined the occurrence of the various E-box motifs within MYCN binding sites that were found exclusively in the *MYCN* amplified state. As displayed in Figure 2C, we observed a significant enrichment of additional motifs CATTTG, CATCTG, CAACTG, potentially indicating that MYCN binding becomes less specific when it is highly abundant. A similar enrichment was observed when plotting the frequency of E-box motifs against raw fluorescent ratios in the Kelly (Figure 2D) and SK-N-AS (Figure S3) NB cell lines.

### Putative MYCN Function in Neuroblastoma

In order to identify sets of biological processes regulated by MYCN, Gene Ontology (GO) analysis [20], [21] was performed using the subset of 265 sites common to all NB cell lines. A total of 270 genes map within a region 3 kb upstream and 1 kb downstream of these 265 MYCN peaks. This group of genes was significantly enriched for genes involved in mRNA transcriptional regulation (n = 121; *p*<0.005), particularly for KRAB box transcription factors (n = 84; *p*<0.002). KRAB-containing proteins are thought to have critical functions in cell proliferation and differentiation, apoptosis and neoplastic transformation [22]. In an effort to determine the aberrant functions of MYCN within cell lines containing the amplification, we generated a data set of genes which were unique to the amplified cell line Kelly when compared to the unamplified SK-N-AS. In a similar manner, we identified genes which were unique to the over-expressing state of the SHEP untreated cell line when compared to the dox treated, low-expressing state.

Gene ontology analysis of genes unique to the MYCN over-expressing states reveals statistically significant enrichment for genes which function across seventeen functional categories and include mRNA transcriptional regulation (Kelly, *p* = 1.18 E-17; SHEP untreated, *p* = 1.41 E-21), DNA helicases (Kelly, *p* = 1.64 E-09; SHEP untreated, *p* = 3.22 E-05), Non-receptor serine/threonine protein kinases (Kelly, *p* = 4.33 E-07; SHEP untreated, *p* = 3.72E-18) and genes involved in G-protein mediated signalling (Kelly, *p* = 2.24 E-09; SHEP untreated, *p* = 1.15 E-12), (Table S2). KEGG pathway analysis revealed a shortlist of genes whose promoters uniquely bound MYCN in the over expressing state and who are members of established signalling pathways which are dysregulated in cancer. Examples include members of the MAPK signaling pathway, such as *NRAS*, a known oncogene; and members of the WNT signaling pathway, *DVL2, DVL3, APC* and *TCF7* which have been previously implicated in colorectal and other cancers [23], [24].

### Co-Localization of MYCN Binding Sites and DNA Hypermethylation

Increasing evidence for MYC family members having an epigenetic role has been previously reported. This includes MYCN exerting a more global effect on transcription by influencing states of chromatin structure [11], as well as the recruitment of methyltransferase DNMT3a to the c-MYC-MIZ1 complex [25]. In order to investigate further the epigenetic link of MYCN function, we carried out methylation profiling of NB cell lines. Hypermethylated DNA was isolated using the methylated DNA immunoprecipitation method – MeDIP [26] and hybridised to a commercial tiling array containing all UCSC annotated CpG islands (28,226) and all known reference gene promoter regions (Promoter Plus Arrays; NimbleGen). Regions shared across the methylation CpG Island/promoter array and the ChIP two-array promoter formats were extracted and used to compare the association of hypermethylation peaks and MYCN TFBS peaks. Regions of MYCN binding and DNA hypermethylation were defined as “associated” only if their mapped genomic coordinates directly overlapped. Table 1 displays the co-occurrence of MYCN binding and DNA hypermethylation across cell lines. The co-occurrence frequency is significantly higher than expected given the individual background frequencies of MYCN binding and hypermethylation in all NB cell lines examined. Furthermore we observed similar E-box usage across both methylated and non-methylated sites on promoter arrays. A pair-wise comparison of SHEP treated and untreated log_2_ ratio methylation data displayed a high degree of correlation (Figure S4; *r* = 0.89), similar to that of biological replicate experiments, indicating that DNA methylation patterns remain relatively stable following the decrease in MYCN levels in this cell line model.

**Table 1:** Association of MYCN transcription factor binding sites and hypermethylated regions in gene promoters.

NB cell line	No. MYCN binding sites	No. hyper-methylated regions	No. MYCN and Hypermethylated regions	MYCN and Hypermethylated regions (%)	No. MYCN and Non-methylated	MYCN and Non-methylated (%)	Observed freq	Expected	P-value
Kelly	960	804	188	19%	772	81%	0.009	0.002	<1e-16
SK-N-AS	640	924	120	19%	520	81%	0.006	0.001	<1e-16
SHEP DOX+	791	1235	215	27%	576	72%	0.010	0.002	<1e-16
SHEP DOX-	1313	1913	418	31%	895	67%	0.020	0.006	<1e-16

To test whether the association of MYCN at sites of hypermethylation is correlated with reduced expression of genes, we have analyzed the Kelly and SK-N-AS cell lines using the NimbleGen expression microarray platform. First, we determined if hypermethylation in the absence of MYCN binding was associated with reduced expression in our data set. A total of 360 genes were identified as differentially methylated between the two cell lines (i.e. methylated in both replicates of one cell line, but not in both replicates of the other) and did not have MYCN binding. From this list, 218 genes were significantly differentially expressed between the two cell lines (1.5 fold minimal difference; Tables S3 and S4). Overall, hypermethylation correlated with lower gene expression for the majority of genes in both Kelly and SK-N-AS (p<0.001). Given that DNA methylation had a significant impact on gene expression in our data set, we then determined if MYCN binding at sites of hypermethylation had an effect on gene expression. We identified 28 genes which were exclusively hypermethylated and MYCN bound in Kelly compared to SK-N-AS. In total, 16 genes displayed differential expression, with 13 of these genes over expressed in Kelly compared to SK-N-AS (p = 0.01; 1.5 fold minimal difference; Table S5). A further analysis identified 29 genes that were hypermethylated in both Kelly and SK-N-AS which also had MYCN binding in Kelly but not in SK-N-AS. Of these, 17 genes were significantly differentially expressed between the two cell lines (Table S6), with a significant bias towards over expression in Kelly (15 up regulated and 2 down regulated; p = 0.002).

**Figure 3:**
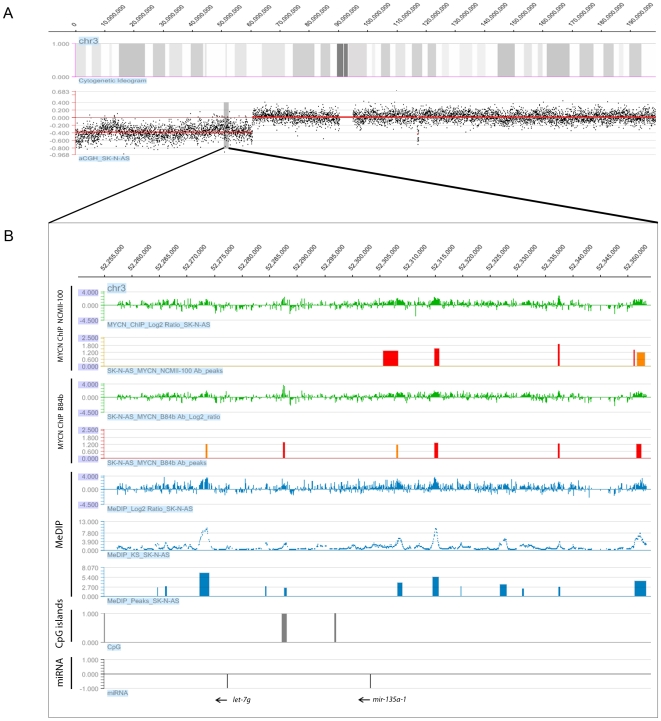
Association of MYCN binding and DNA hypermethylation around the let 7g and miR-135a-1 locus in SK-N-AS NB cell line. (A) Array-CGH profile of chromosome 3p displaying a large 60.5 Mb terminal deletion in SK-N-AS. (B) Detailed view of the hemizygously deleted let-7 g/miR135a-1 locus. The upper two panels display the MYCN ChIP-chip raw log_2_ ratios and identified peaks for both MYCN antibodies (NCMII-100 and B84b). Red and orange peaks represent regions of enrichment with an FDR of <0.05 and 0.05–0.1, respectively. MeDIP results for SK-N-AS are displayed in the lower panels in blue, which include log_2_ ratios of MeDIP/Input, Kolmogorov-Smirnov test p-values (−log_10_) and the merged statistically significant peaks of hypermethylation across the region. The position of CpG islands and orientation of miRNAs are displayed in the two bottom panels.

The relationship between MYCN binding and hypermethylation beyond promoter regions was also investigated using a custom tiling array (see methods). As this array consists of non-discrete tiled regions, the association of log_2_ ratios from MYCN ChIP-chip and MeDIP-chip was evaluated using Pearson's correlation and its corresponding p-value. Results based on this analysis confirmed that the correlation between MYCN binding and DNA hypermethylation extended to intergenic regions tiled on the arrays (Table 2). Selected methylated loci were confirmed by sequencing of bisulfite treated DNA (Table S7). In order to determine if this association occurs on the same copy of the chromosome we determined DNA copy number status on each cell line using microarray-based comparative genomic hybridisation (aCGH). Figure 3 displays the co-localization of MYCN binding sites with sites of DNA hypermethylation within a hemizygously deleted region on chromosome 3 in SK-N-AS. The detailed panel 3B displays an example of this combined MYCN/DNA hypermethylation analysis across 100 kb of genomic sequence encompassing the let-7 g and miR-135a-1 loci, illustrating the high correlation between MYCN binding and sites of DNA hypermethylation. Reverse transcriptase qPCR assays indicated that both of the miRNAs shown in Figure 3 are expressed in SK-N-AS (data not shown). However, the relationship of the identified peaks of hypermethylation/MYCN binding with expression is complicated by the fact that very little is known about the miRNA promoters. Of the four studies [27]-[30] which have reported on this subject, only Marson *et al*. [28] have reported a putative miRNA promoter region for mir-135a (chr3: 52307870-52308065) and 2 studies (Marson & Ozsolak *et al*.) [28], [29] have identified a putative let-7g promoter (region common to both studies; chr3:52207870-5230865). Both putative miRNA promoter regions fall outside of our identified peaks of hypermethylation and MYCN binding in SK-N-AS. The transcriptional control of let-7g is further complicated by the fact that it is present within the second intron of the WDR82 gene.

**Table 2:** Association of MYCN transcription factor binding sites and hypermethylated regions in inter/intragenic regions.

NB cell line	No. MYCN binding sites	No. hypermethylated regions	No. MYCN and Hypermethylated regions	MYCN and Hypermethylated regions (%)	Pearson's correlation (*r*) of log_2_ ratios	P-value
SK-N-AS	638	3148	487	76%	0.608	P<10e-16
SHEP DOX+	411	2982	260	63%	0.535	P<10e-16
SHEP DOX-	1188	3089	677	57%	0.503	P<10e-16

## Discussion

The *MYCN* oncogene was originally identified due to its frequent amplification and over expression in certain tumours, chiefly human neuroblastoma [31], [32]. In this study, we have identified and analysed high confidence MYCN binding sites using ChIP-chip assays based on two independent antibodies to gain an insight into the binding patterns and possible functions of MYCN. As far as we are aware, this conservative strategy has not been applied to ChIP-chip analysis of other transcription factors.

### MYCN Exhibits Significant Selection of the CATGTG/CACATG E-box Motif

It has previously been reported that both c-MYC and MYCN bHLH transcription factors bind to the canonical E-box motif CAC(A/G)TG [33]-[36]. More recent studies have suggested that c-MYC exhibits a significant preference for CACGTG and a negative selection for CATGTG [37]. Our results indicate that MYCN exhibits greater E-box selectivity for the CATGTG motif in NB cell lines than for the classic CACGTG motif. Interestingly, in a recent MYCN ChIP analysis of the *LIF* promoter, which contains both CACGTG and CATGTG, exclusion of the CATGTG sequence resulted in a significant reduction of MYCN binding *in-vitro* [17]. The greater affinity of MYCN for CATGTG appears to occur in both MYCN amplified and non-amplified cells. Our findings are also consistent with the analysis of genes which are differentially expressed in response to MYCN over expression in NB cell lines, where the CATGTG motif was identified in the promoter regions of 95/139 differentially expressed genes (68%) compared to the CACGTG motif which was detected in promoter regions of 41/139 genes (29%) [4]. In contrast to our results, Cottermann *et al* previously reported a statistically significant over representation for the CACGTG E-box only [11]. However, this study varied in its experimental approach (e.g., use of one antibody, microarray design and data analysis parameters) and the reported enrichment analysis was based on a comparison between the presence of the CACGTG motif and all other E-box variants grouped together as CA*NN*TG. Differences in reported E-box frequencies between both studies may therefore exist as we analysed each distinct class of E-box within the CA*NN*TG group. This approach avoided any possible masking effect which an under represented E-box (CATTTG, Figure 2B) might have over a significantly over represented E-box (CAACTG, Figure 2B) within the CA*NN*TG group.

*In-vitro* analyses have previously demonstrated the binding of c-MYC to cis-acting canonical E-boxes CACGTG and CATGTG [36] as well as non-canonical variants. Later ChIP-seq *in-vivo* analysis indicated that c-MYC bound to both forms in B-cells [19] and that the CACGTG was predominant in HeLa cells. Previous evidence indicates some E-box preference in the MYC family where Kim *et al.* demonstrated that although CACGTG is enriched, an increase in Myc binding to promoters that contain each of the previously defined E-box sequences, except CATGTG was observed [37].

From the functional perspective, Westermann *et al.* [13] reported an inverse relationship between MYCN and c-MYC expression in different neuroblastoma tumor subtypes, where c-MYC was expressed at higher levels in *MYCN* single copy high risk tumors relative to *MYCN* amplified or *MYCN* single copy low risk tumors. Although this finding indicated a potential redundancy for the functions of these genes in tumorigenesis, the authors also demonstrated that a significant number of MYCN/c-MYC target genes are less responsive to MYCN than to c-MYC and also that a subgroup of genes were repressed by MYCN but not by c-MYC. Our results, highlighting differences in E-box selection between MYCN and c-MYC would indicate that these transcription factors should not be completely functionally redundant in cancer. The differences in E-box usage also have relevance to the role of these genes in normal developmental processes. Both proteins clearly play distinct roles in development since homozygous null c-Myc and *MYCN* mice die at about embryonic days 10 and 12, respectively. When *mycn* is inserted into the c-*myc* locus in mice, it is expressed and regulated similarly to c-myc during development and to a great degree restores normal development to c-myc homozygous null mice [38]. However, non-redundancy is evidenced by the fact that these mice are significantly smaller and in some cases display dystrophy of skeletal muscle. This evidence suggests that although mycn and c-myc share some functional roles, *mycn* cannot completely replace all functions of c-myc even when similarly regulated. The fact that these proteins share only 32% amino acid sequence similarity [39] is another good indication that they should not be completely functionally redundant. Tissue specific conditional *mycn* knockout in neural progenitor cells during mouse development has demonstrated the importance of mycn for regulating neurogenesis [40], perhaps explaining the greater role of MYCN in neuroblastoma pathogenesis over c-MYC.

### Potential Oncogenic Functions of MYCN in the Amplified State

Amplification of the MYCN locus (up to 200 copies) in NB leads to increased protein production and based on our results, leads to an extra 1,142 MYCN peaks detected within promoter regions in Kelly compared to the *MYCN* unamplified cell line SK-N-AS. Gene ontology analysis of targets which are uniquely bound by MYCN in the over expressing state revealed enrichment for DNA helicases and mRNA transcriptional regulation, amongst others. It has been previously shown that MYCN may mediate proliferation in NB through association with the promoter regions of the minichromosome maintanence complex genes (MCM), resulting in increased expression [8]. The MCM protein family function as DNA helicases which are critical for DNA synthesis, play a significant role in various aspects of genome stability [41], [42] and are dysregulated in numerous cancer types [43]–[46]. However, the role of the specific MYCN positive DNA helicases and other functional groups identified remains the subject of further study.

A more focused KEGG pathway analysis of our data indicates that MYCN binds upstream to a number of genes in the WNT signalling pathway. Dysregulation of genes associated with the WNT/β-Catenin pathway has been previously reported in a cohort of 73 primary NB tumours [47]. Interestingly, differential expression of genes within the pathway was identified between MNA and high-stage non-MNA subtypes. In our data, *DVL2*, *DVL3*, *APC* and *TCF7* were identified as bound by MYCN only in the over expressing state (Kelly and SHEP untreated). However, these targets must be further functionally tested to evaluate the influence of MYCN on this pathway. Finally, additional genes which were identified from our MYCN binding analysis have also been recently reported by Vermeulen *et al.* [48] as part of a 59-gene expression signature which was applied as an accurate predictor of clinical outcome in patients with neuroblastoma. Although multivariate logistic regression analysis showed that the signature was independently statistically significant in a model adjusted for *MYCN* status and other predictors of prognosis, we have determined that a number of these genes including *PTN* and *CPSG3*, have positive MYCN binding sites upstream suggesting that MYCN may play a part in the control of these genes.

Based on the analysis of E-box usage in the *MYCN* amplified state, we hypothesize that over expression of MYCN in the amplified state may lead to aberrant binding to additional weaker affinity E-box motifs such as CATTTG, CATCTG and CAACTG. One striking observation when comparing the E-box usage of MYCN targets in the unamplified compared to the amplified state was that one motif, CATTTG, switched from being statistically underrepresented in the unamplified state (*p* = 0.014) to statistically significantly over represented in the amplified state (*p* = 0.023). Interestingly, a bHLH transcription factor which is phylogenetically related to MYC, microphthalmia-associated transcription factor (MITF) [49], binds the classic E-box motif CACGTG as well as CATTTG [50]-[52]. MITF, which is not expressed in NB cells [53], has been shown to have a role in the regulation of melanocyte development and is similar to MYCN in that it is a reported oncogene which is amplified in cancer [54]. It may be possible that an excess of MYCN in *MYCN* amplified NB leads to weak aberrant binding at unoccupied CATTTG sites potentially deregulating the expression of MITF or other bHLH targets in NB cells, thus promoting tumorigenesis.

### Associated MYCN and Hypermethylated Loci

Utilizing a combined genome-wide ChIP-chip/MeDIP-chip approach we demonstrated that MYCN binding is highly enriched in genomic regions of DNA hypermethylation and that enrichment of this co-localization is prominent within intra- and intergenic regions in addition to promoter sequences. Previous promoter based *in-silico* analysis of hypermethylated DNA sequences has demonstrated enrichment for specific transcription factors [55]. The non-random association of MYCN binding with DNA hypermethylated sequences might be explained through the previous observation that c-MYC can recruit the DNA methytransferase DNMT3a to the promoter region of genes to induce gene silencing [25]. Brenner *et al.* [25] determined that there was a synergistic repressive effect on the promoter of p21, when both c-MYC and DNMT3a were co-transfected into U2OS cells. The model involved c-MYC binding to the cofactor MIZ to form a ternary complex with DNMT3a at the promoter of p21, inducing methylation. Although, structural differences exist between c-MYC and MYCN within the N-terminal domain, both proteins contain the MBI and MBII domains which are required to associate with DNMT3a [25], [56], indicating that MYCN could interact in a similar manner.

Interestingly, Perini *et al*. [12] demonstrated that methylation of the CpG dinucleotide within the CACGTG E-box prevents MYCN binding to a number of gene promoters [12]. Our results showing preferential association of MYCN to sites that are hypermethylated are not inconsistent with Perini *et al.* since our array results are not of sufficient resolution to ascertain the methylation status of specific base pairs. In addition, analysis of E-box motifs confirmed that there was no significant difference in the E-box usage of the classical CACGTG in methylated compared to non-methylated sites, which one would expect if this sequence was predominantly utilised by MYCN. It is also possible that in many instances MYCN is not binding directly to DNA sequences, but instead is interacting with another DNA binding partner, such as a methyl binding protein.

Through the integration of DNA copy number, MeDIP-chip and MYCN ChIP-chip data for the same NB cell lines we have identified overlapping regions of hypermethylated DNA and MYCN binding within hemizygously deleted chromosomal regions, thus confirming that this association occurs on the same allele. Previously, Cotterman *et al*. [11] determined that MYCN is a weak transcription factor even at genes which it directly binds and found that overall changes in the expression of MYCN bound genes were modest, most often in the range of 2-fold or less despite profound chromatin modification changes. Based on our expression analysis of methylated genes, we confirmed that differentially hypermethylated genes correlated with lower expression in both NB cell lines studied. Surprisingly, upon integrating the MYCN ChIP-chip, methylation and expression data, we discovered that genes which are MYCN bound and hypermethylated tend to have increased expression compared to genes which are methylated/not MYCN bound or not methylated/not MYCN bound. The association of MYCN with higher expression of methylated promoter regions although interesting, requires further experimentation to determine if there is an actual cause and effect relationship. In addition, other factors such as differences in miRNA expression profiles could also be influencing gene expression.

It has also been reported that MYCN has a much more global role in the regulation of transcription and chromatin than previously anticipated and that it is required to maintain euchromatin in a wide spread manner, including at intergenic sites [11], [57]. When analysed, the association of putative MYCN binding sites within inter- and intragenic regions was more prominent than that observed at annotated classical promoter sequences, possibly supporting its role as a more global mediator of chromatin modification. Our results further support a possible dual role for MYCN and DNA hypermethylation, namely that of a classical transcriptional repressor of upstream genes and that of a mediator of global chromatin structure. A future aspect which should be considered is a more comprehensive analysis of MeDIP, MYCN ChIP-chip and other possible co-factors such as DNMT3a, using sets of higher density whole genome arrays or a next generation sequencing platform.

## Materials and Methods

### Western Blot

Total cell lysate and nuclear extractions were prepared from the cultured neuroblastoma cell lines SHEP-21N, Kelly and SK-N-AS. These lysates were added to Laemmlibuffer, electrophoresed in a 10% SDS-PAGE gel and transferred to a nitrocellulose membrane. After blocking overnight with milk protein, the blots were probed with either a 1 in 500 dilution of NCMII-100 (ab16898; Abcam) or B84b (sc-53993; Santa Cruz Biotechnology) antibodies, followed by a 1 in 2000 dilution of secondary rabbit anti-mouse IgG antibody (ab6728-1; Abcam). The specificity of the antibodies for MYCN was confirmed and blots were then stripped and reprobed using a 1 in 2000 dilution of mouse anti-GAPDH antibody (Ab9484; Abcam). The expression of MYCN was detected in all cell lines (Figure S1C–E).

### Cell Culture

SHEP-21N Cells were obtained from Dr Louis Chesler with the permission of Prof. Manfred Swab. The Kelly and SK-N-AS cell lines were obtained from the European Collection of Animal Cell Cultures (Porton Down, United Kingdom). SK-N-AS cells were grown in EMEM supplemented with 1% non-essential amino acids, 2 mM L-glutamine, 10% fetal bovine serum and penicillin/streptomycin. Kelly cells were cultured in RPMI-1640 supplemented with 2 mM L-glutamine, 10% fetal bovine serum and penicillin/streptomycin. SHEP-21N cells were grown in RPMI 1640 with 2 mM L-glutamine, penicillin/streptomycin and 10% tetracycline-free fetal bovine serum (HYC-001-333C, Fisher Scientific) In order to abrogate the expression of MYCN in the SHEP-21N cell line, the cells were maintained in their growth media supplemented with 50ng/ml Doxycycline (8634-1, Clontech). Cells were cultured using Hyperflask cell culture vessels (#10010, Corning) for six days. All cell culture reagents were obtained from GIBCO unless otherwise stated.

### ChIP-Chip Analysis

ChIP assays were performed using the above antibodies according to the standard NimbleGen ChIP protocol. Briefly, 1×10^9^ cells were fixed with 1% formaldehyde solution for 10 min on ice, centrifuged and rinsed with ice-cold PBS. Cell nuclei were isolated and sonicated to generate DNA fragments of approximately 1 kb in length. The resulting fragmented chromatin was aliquoted into 2 mg/ml stocks and stored at −80°C. One 2 mg/ml aliquot was used per ChIP reaction. DNA was enriched by immunoprecipitation using 10 µg of either the B8.4.B or NCMII-100 anti-MYCN antibodies complexed to M-280 Sheep anti-Mouse Dynabeads (112-02D, Invitrogen). The formaldehyde crosslinks, protein and RNA was removed from the immunoprecipitated DNA sample through heat denaturing, proteinase K and RNase A treatments. An un-enriched sample of DNA was treated in a similar manner to serve as input. ChIP and input DNA was fluorescently labelled using Klenow fragment (M0212M, New England Biolabs) and Cy5/Cy3 random primers (N46-0001-50/N46-0002-50, TriLink BioTechnologies). The Cy5-ChIP and Cy3-input labelled DNA samples were co-hybridized to microarrays for 18 hours, post hybridisation washes were carried out and microarrays were scanned using an Axon 4000B microarray scanner with GenePix 6.0 (Molecular Devices). Microarrays used included the HG18 two-Array Promoter Set and a custom miRNA array manufactured by Roche NimbleGen. The promoter arrays include an average coverage of 4.7 kb around promoters for all RefSeq genes, UCSC known genes and the Mammalian Gene Collection. A custom array was designed to include tiled sequence of 50 kb 5′ and 20 kb 3′ of 528 miRNAs. Image files generated after scanning were analyzed using NimbleScan Software Version 2.4. Sites of enrichment were identified using the normalised log_2_ ratios and the NimbleScan peak finding function. The in-built peak analysis algorithm detects significantly enriched regions that have at least 4 probes above a threshold value of log 2.0, identifies them as “peaks” and assigns a false discovery rate (FDR). An FDR value of 0.1 was used in the initial screening of peaks from individual experiments for both NCMII-100 and B84b antibodies. Peaks with an FDR of less than 0.1 which were shared across ChIP reactions using both MYCN antibodies were filtered using an in-house *Java* application. This produced a final set of consistently bound high confidence regions for each of the cell lines used in the study. This final set of high confidence peaks was subsequently used in further transcription factor binding site and Gene Ontology analysis.

### Transcription Factor Binding Site Analysis

DNA sequence data for the processed ChIP regions for each cell line were retrieved from the UCSC database [58]. Phylogenetically conserved sequence between Human (Hg18) and Mouse was selected for motif analysis. E-box specificity was assessed by examining the occurrence of the various forms of E-boxes conforming to the generic CA*NN*TG motif. When counting E-box motifs within the ChIP derived putative MYCN binding sites, both the forward and reverse of each motif was taken into account. If this were not the case palindromic sequences such as CACGTG would be artificially over-represented with respect to other motifs. The sequence present on the promoter array was used to assess the background frequency of each E-box motif. In order to assess over or under-representation within ChIP sequences it is also essential to take into account the lengths of the ChIP sequences. For example, if the average length of a ChIP pull-down is 550 bp this window must also be used when performing a random background count of this motif. Significance for over (or under) -representation was assessed using P-values based on Fisher's Exact test when compared to background motif frequency.

### Gene Ontology Analysis

Gene Ontology analysis was carried out using the DAVID Functional Annotation Tool [20], [21]. In all analysis the following parameters were used - Functional enrichment was assessed using the Panther Biological Process and Molecular Function and KEGG Pathway databases. A custom *Java* based application was used to identify common and unique peaks of enrichment across cell lines and the resulting gene Entrez IDs were submitted to DAVID. Significance of over-representation of functional categories was assessed via Fisher's exact test and corrected for multiple comparisons using the Bonferroni method. After analysis, all categories with a *p* value greater than 0.001 and which represented less than 5% of the total number of genes was eliminated. Redundant and non-informative terms (eg. Other Metabolic Pathways) were also excluded.

### Q-PCR Analysis

Taqman Probes were designed and manufactured by Applied Biosystems against regions of enrichment identified for the ChIP-chip experiments. A negative control probe was also designed against un-enriched genomic regions for comparison. Q-PCR analysis was performed on immunoprecipitated and un-enriched input DNA samples in duplicate. The relative level of enrichment (RQ) was calculated for each of the target regions using the comparative Ct method. Details of primer and probe sequences are provided in Table S8.

### Methylated DNA Immunoprecipitation

The protocol used was as previously described by Weber *et al.* [26]. Briefly, five micrograms of DNA was fragmented by sonication to 400–800 bp in size and confirmed by running 100 ng on a 2% agarose gel. Four micrograms of the sonicated DNA was incubated overnight with an anti-5′ methyl-cytidine antibody (BI-MECY-1000; Eurogentec). A Taqman quality control qPCR assay was performed to detect the relative fold change of enrichment of the methylated *H19* locus relative to an unmethylated *H3B* [59], prior to microarray hybridization (Figure S5). The MeDIP DNA and reference control were differentially labelled using Cy5 and Cy3 respectively, and co-hybridised to a custom miRNA array and the CpG Island promoter plus array from Roche NimbleGen. The 385,000 probe CpG Island Plus Promoter Array includes all UCSC-annotated CpG islands, 1 kb tiled sequence around all RefSeq gene promoter regions and DNA methylation positive control regions (*HoxA, H19/IGF2, KCNQ1* clusters). Arrays were scanned using the GenePix 4000B scanner and the following analysis was performed using the Nimblescan Software Version 2.4: normalised log_2_ ratio data was calculated and a one-sided Kolmogorov-Smirnov test (KS; using a sliding window of 750 bp) was applied to determine whether the probes were drawn from a significantly more positive distribution of intensity log-ratios than those on the rest of the microarray. The resulting score for each probe was the -log_10_ p-value from the windowed KS test around that probe. Hypermethylated peaks were detected by searching for at least 2 probes above a p-value minimum cut off (−log_10_ of 2) and peaks within 500 bp of each other are merged. Resulting data files were visualised using SignalMap 1.9. Experiments were performed in duplicate. Pair-wise comparison of cell line replicates resulted in high correlation co-efficients [*r* = 0.82 (Kelly) and 0.89 (SK-N-AS); Figure S6]. Peaks only detected in both experiments were used for further analysis.

### Bisulphite Sequencing

A total of 500 ng of DNA from Kelly and SK-N-AS was bisulphite converted using the EZ DNA-methylation Gold kit (Cat. No. D5005 & D5006, Zymo) using the alternative conversion reaction 2 as per manufacturer's instructions. The converted DNA was subsequently purified in 50 µl of elution buffer. PCR primers were designed using methyl primer express (www.appliedbiosystems.com/methylprimerexpress). PCR was carried out using AmpliTaq Gold mastermix (Cat.No. 4326717, Applied biosystems) under the following conditions: 95°C for 10 min; 94°C for 30 sec, 58–60°C for 30 sec, 72°C for 30 sec (35 cycles) followed by 72°C for 10 min. The PCR product was resolved on a 1.5% agarose gel with SYBR-safe. Twenty microlitres of PCR product was subsequently purified using ExoSAP-IT (Cat No. 78200, USB) and QIAquick PCR purification kit (Cat. No. 28104, Qiagen), as per manufacturer's instructions. Purified PCR product was further sequenced in the forward and reverse direction at MWG biotech. Resulting electropherograms were analyzed using the BIQ analyzer [60].

### Array-CGH Analysis

Array CGH was carried out as previously described [61] using a 385,000 feature array from NimbleGen.

### Gene Expression Analysis

Total RNA was extracted from Kelly and SK-N-AS using the RNeasy Mini kit (Cat. No. 74104, as per manufacturer's instructions QIAGEN), including on-column digestion of DNA using the RNase-Free DNase Set (Cat. No. 79254, QIAGEN), to ensure complete DNA removal. RNA integrity was confirmed using the Experion RNA StdSens Analysis Kit (Cat. No. 700-7103, Bio-Rad). The ExpressArt TR Micro Kit (Cat. No. 6199-A30, AmpTec) was used to synthesise double-stranded cDNA from 3 ug total RNA, which was subsequently used to generate amplified amino-allyl antisense RNA (aRNA) using the SuperScript Indirect RNA Amplification System (Cat. No. L1016-01, Invitrogen). The aRNA was coupled to Cy3 reactive dye (Cat. No. 25-8010-79, Amersham Biosciences). Four micrograms of Cy3-aRNA was hybridised to the Homo sapiens 4x72K Gene Expression Array from Roche NimbleGen (Cat. No. A4487001-00-01), as per manufacturer's instructions. Arrays were scanned using the GenePix 4000B scanner and gene expression data analysis was performed using NimbleScan Software Version 2.4.

## Supporting Information

Figure S1 Performance of MYCN ChIP-chip antibodies. (A & B) Pair-wise comparison of log_2_ ratio between two different antibodies used for the ChIP reactions on microarrays containing promoter regions from chromosome 1 to 10p (A) and chromosome 10q to Y (B). An average value over 4 probes was used for plotting, consistent with the peak finding analysis software criteria. Pearson correlations (r) is displayed at the top right of each panel. (C) Western blot of Kelly and SHEP nuclear extracts using the MYCN antibody NCMII-100, (D) Western blot of Kelly and SHEP and SK-N-AS nuclear extracts using the MYCN antibody B84b. (E) Western blot of SK-N-AS using both MYCN antibodies. Blots were reprobed for GAPDH which confirmed even loading across wells.
(0.73 MB TIF)

Figure S2 PCR validation of MYCN ChIP reactions. qPCR validation of positive MYCN transcription factor binding sites. Fold enrichment of positive MYCN target sites is displayed. Experiments were carried out in duplicate using the delta-delta Ct method and results are plotted relative to a negative MYCN binding region (SEMA3B) identified on the arrays and set to 1.0.
(0.31 MB TIF)

Figure S3 Association of E-box frequency to raw fluorescent ratios in SK-N-AS. Y-axis represents the E-box frequency per kilobase, while the x-axis indicates the florescent intensity ratios.
(0.08 MB TIF)

Figure S4 Pair-wise comparison of MeDIP log_2_ ratios between SHEP treated and untreated cell lines. An average value over 4 probes was used for plotting. Pearson correlations (r) is displayed at the top of the panel.
(0.21 MB TIF)

Figure S5 qPCR enrichment of MeDIP reactions. Graph displays fold enrichment for Kelly and SK-N-AS using the Ct method for the imprinted H19 locus versus a non-methylated H3B promoter following immunoprecipitation with an anti-methyl cytidine antibody, as used by Weber et al. [59]. A negative control MeDIP reaction using an isotype matched normal mouse IgG antibody is also displayed. PCR reactions were performed in duplicate.
(3.18 MB TIF)

Figure S6 Pair-wise comparison of MeDIP log_2_ ratios between NB cell line replicates. (A) Kelly and (B) SK-N-AS pair-wise comparison plots. An average value over 4 probes was used for plotting. Pearson correlations is displayed at the top of the panel.
(0.49 MB TIF)

Table S1 MYCN binding sites common to all NB cell lines.
(0.09 MB PDF)

Table S2 Gene ontology of MYCN target genes.
(0.07 MB PDF)

Table S3 Expression of genes (fold change <0.5 and >1.5) which are methylated in SK-N-AS and not methylated in Kelly.
(0.07 MB PDF)

Table S4 Expression of genes (fold change <0.5 and >1.5) which are methylated in Kelly and not methylated in SK-N-AS.
(0.07 MB PDF)

Table S5 Expression of genes (fold change <0.5 and >1.5) which are methylated and MYCN bound in Kelly and not methylated or MYCN bound in SK-N-AS.
(0.04 MB PDF)

Table S6 Expression of genes (fold change <0.5 and >1.5) which are methylated and MYCN bound in Kelly and methylated but not MYCN bound in SK-N-AS.
(0.03 MB PDF)

Table S7 Bi-sulphite sequencing of selected loci.
(0.01 MB PDF)

Table S8 Taqman q-PCR primer and probes.
(0.06 MB PDF)
