# Supplementary figures and images for: Global MYCN Transcription Factor Binding Analysis in Neuroblastoma Reveals Association with Distinct E-Box Motifs and Regions of DNA Hypermethylation
Source: PLoS One. 2009 Dec 4;4(12):e8154. doi: 10.1371/journal.pone.0008154 (PMC2781550; doi:10.1371/journal.pone.0008154)

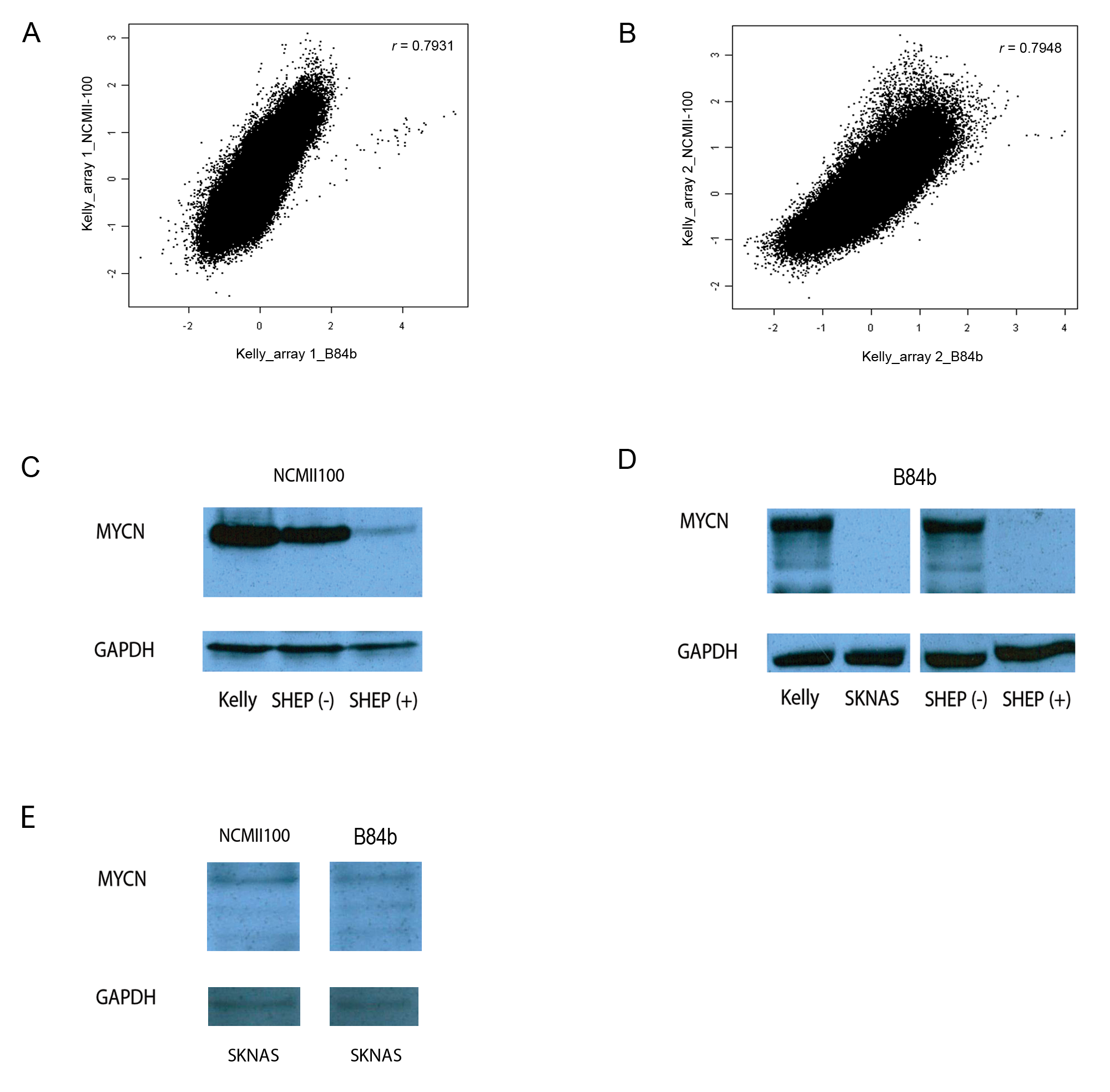

Supplement: Figure S1 — Performance of MYCN ChIP-chip antibodies. (A & B) Pair-wise comparison of log2 ratio between two different antibodies used for the ChIP reactions on microarrays containing promoter regions from chromosome 1 to 10p (A) and chromosome 10q to Y (B). An average value over 4 probes was used for plotting, consistent with the peak finding analysis software criteria. Pearson correlations (r) is displayed at the top right of each panel. (C) Western blot of Kelly and SHEP nuclear extracts using the MYCN antibody NCMII-100, (D) Western blot of Kelly and SHEP and SK-N-AS nuclear extracts using the MYCN antibody B84b. (E) Western blot of SK-N-AS using both MYCN antibodies. Blots were reprobed for GAPDH which confirmed even loading across wells. (0.73 MB TIF) [file pone.0008154.s001.tif]

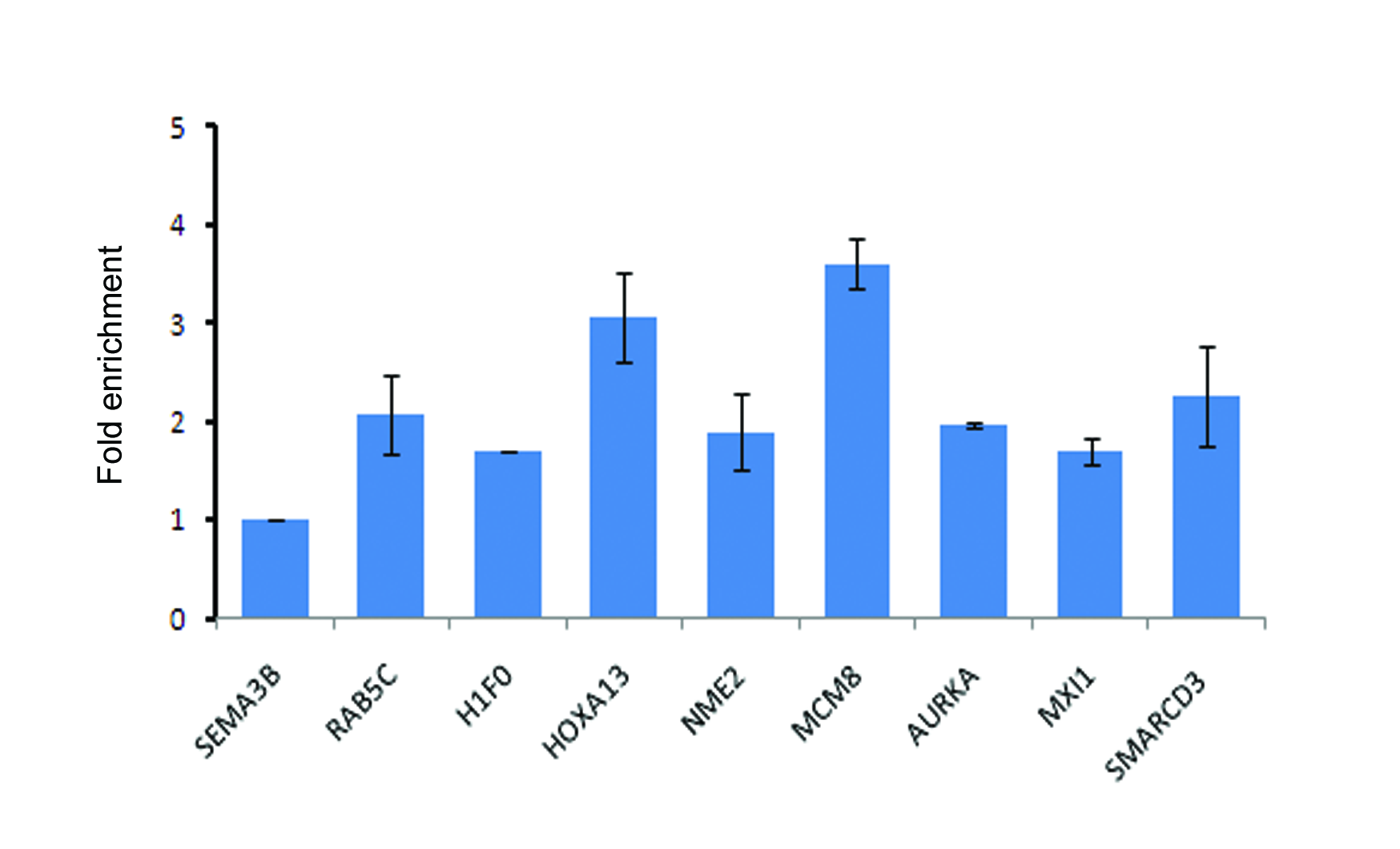

Supplement: Figure S2 — PCR validation of MYCN ChIP reactions. qPCR validation of positive MYCN transcription factor binding sites. Fold enrichment of positive MYCN target sites is displayed. Experiments were carried out in duplicate using the delta-delta Ct method and results are plotted relative to a negative MYCN binding region (SEMA3B) identified on the arrays and set to 1.0. (0.31 MB TIF) [file pone.0008154.s002.tif]

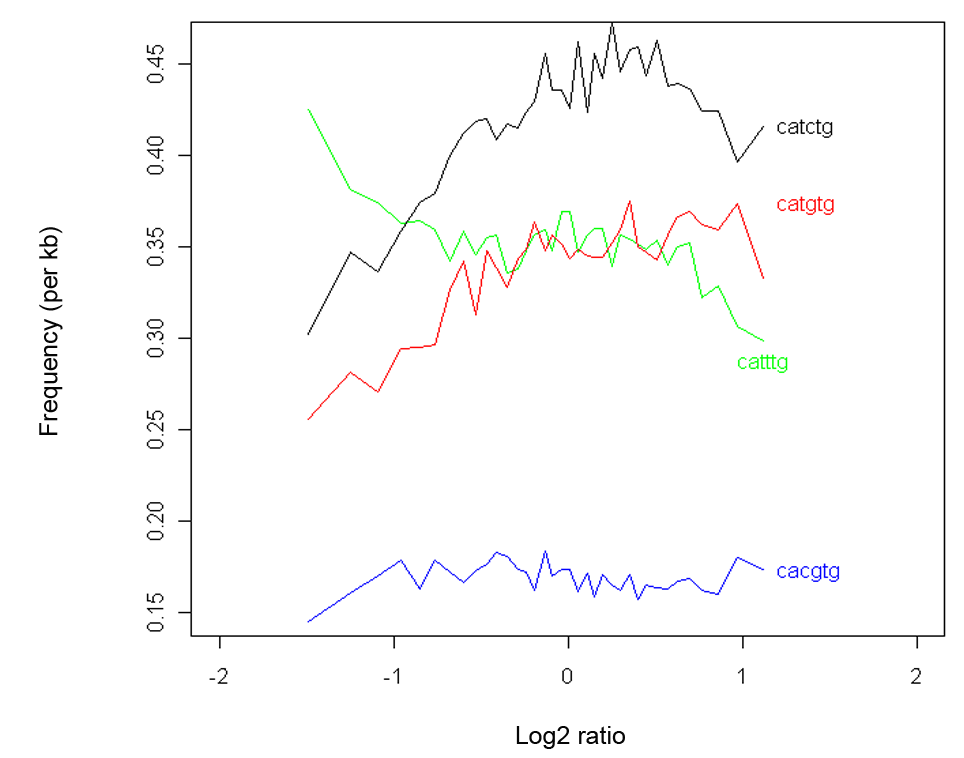

Supplement: Figure S3 — Association of E-box frequency to raw fluorescent ratios in SK-N-AS. Y-axis represents the E-box frequency per kilobase, while the x-axis indicates the florescent intensity ratios. (0.08 MB TIF) [file pone.0008154.s003.tif]

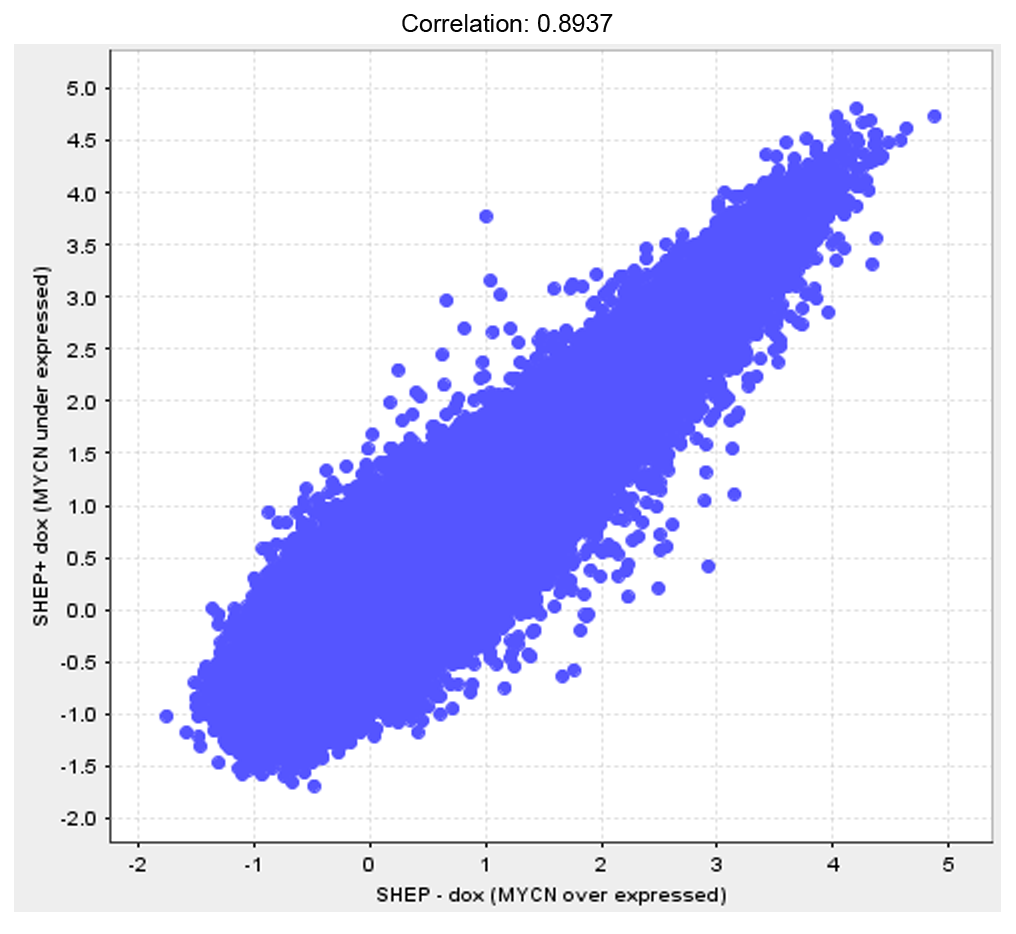

Supplement: Figure S4 — Pair-wise comparison of MeDIP log2 ratios between SHEP treated and untreated cell lines. An average value over 4 probes was used for plotting. Pearson correlations (r) is displayed at the top of the panel. (0.21 MB TIF) [file pone.0008154.s004.tif]

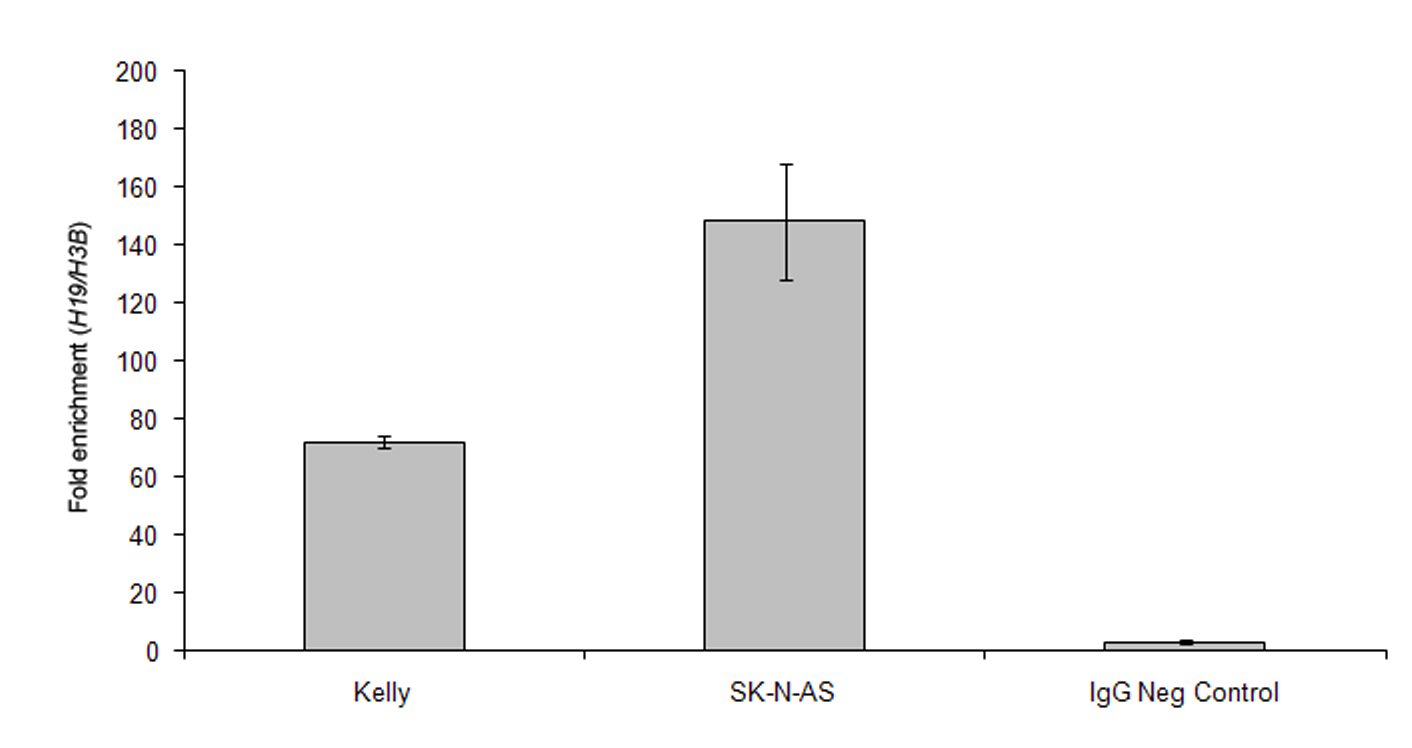

Supplement: Figure S5 — qPCR enrichment of MeDIP reactions. Graph displays fold enrichment for Kelly and SK-N-AS using the Ct method for the imprinted H19 locus versus a non-methylated H3B promoter following immunoprecipitation with an anti-methyl cytidine antibody, as used by Weber et al. [59]. A negative control MeDIP reaction using an isotype matched normal mouse IgG antibody is also displayed. PCR reactions were performed in duplicate. (3.18 MB TIF) [file pone.0008154.s005.tif]

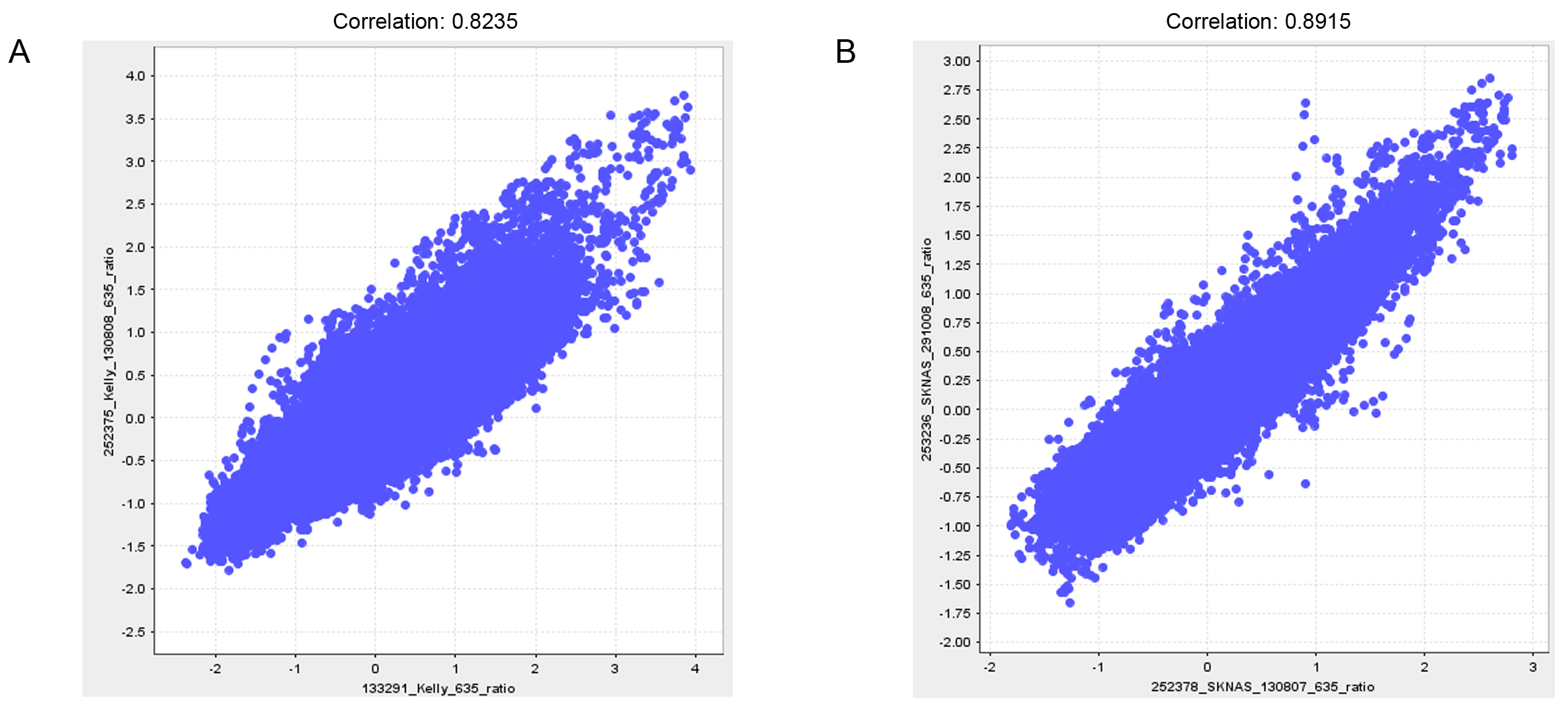

Supplement: Figure S6 — Pair-wise comparison of MeDIP log2 ratios between NB cell line replicates. (A) Kelly and (B) SK-N-AS pair-wise comparison plots. An average value over 4 probes was used for plotting. Pearson correlations is displayed at the top of the panel. (0.49 MB TIF) [file pone.0008154.s006.tif]
