# Supplementary material for: Global MYCN Transcription Factor Binding Analysis in Neuroblastoma Reveals Association with Distinct E-Box Motifs and Regions of DNA Hypermethylation
Source: PLoS One. 2009 Dec 4;4(12):e8154. doi: 10.1371/journal.pone.0008154 (PMC2781550; doi:10.1371/journal.pone.0008154)
Supplement: Table S1 — MYCN binding sites common to all NB cell lines. (0.09 MB PDF) [file pone.0008154.s007.pdf]

Supplementary Table 1. MYCN binding sites common to all NB cell lines

Chromosome	Start position	End position	E-boxes	Nearest Gene	Entrez Gene ID
1	15605	15953	[cacttg, caactg]	*MGC52000*	375260
1	5975869	5976117	-	*NPHP4*	261734
1	11481779	11482127	[catgtg]	*PTCHD2*	57540
1	11953390	11953438	-	N/A	N/A
1	20384465	20384713	[cacttg, catctg]	*UBXD3*	127733
1	22176405	22176454	-	*ELA3B*	23436
1	22201036	22201184	-	*ELA3A*	10136
1	34404003	34404351	[catttg]	*CSMD2*	114784
1	37795505	37795753	[catctg, cacctg, catgtg]	*DNALI1*	7802
1	39752325	39752373	-	*OXCT2*	64064
1	44170979	44171027	-	*ARTN*	9048
1	44206298	44206746	[cacttg, catatg]	*DPH2*	1802
1	46787616	46787764	[catgtg]	*FLJ32011*	148930
1	48460944	48461292	[catctg, catgtg]	*SLC5A9*	200010
1	51535933	51536082	-	*C1orf34*	22996
1	54132892	54133040	-	*DIO1*	1733
1	54784359	54784607	[cacctg]	*ACOT11*	26027
1	87153735	87153983	[catctg, catgtg]	*HS2ST1*	9653
1	120640885	120641033	-	*FAM72A*	389835
1	142623998	142624146	-	*LOC647135*	647135
1	148175818	148175969	[catgtg]	*MTMR11*	10903
1	149585578	149586227	[cacttg, catgtg, cacgtg, catatg]	*RFX5*	5993
1	151222281	151222429	[catctg, cacctg]	*SPRR1A*	6698
1	151865697	151865945	[catatg]	*S100A13*	6284
1	153101694	153102042	[cacttg]	*KCNN3*	3782
1	153270424	153270572	[catttg]	*DCST2*	127579
1	153372450	153372698	[catttg]	*RAG1AP1*	55974
1	153982939	153982987	[cacctg]	*MSTO1*	55154
1	154666465	154666913	[cacttg]	*C1orf61*	10485
1	154855930	154856178	[catatg]	*HAPLN2*	60484
1	155002607	155002755	-	*PRCC*	5546
1	156330773	156331121	-	*KIRREL*	55243
1	157408544	157408692	-	*IGSF4B*	57863
1	157438534	157438882	[cacttg, cacctg, cagctg]	N/A	N/A
1	158061625	158061973	-	*SLAMF8*	56833
1	158137114	158137666	[catttg, cacctg]	*CCDC19*	25790
1	158352472	158352520	-	*ATP1A2*	477
1	158602798	158602846	-	*NHLH1*	4807
1	159282292	159282440	[caactg, cagctg]	*USF1*	7391
1	176776454	176776802	[catttg, catctg, catgtg]	*FLJ35530*	400798
1	180636325	180636373	-	*TEDDM1*	127670
1	180908291	180908546	[catttg, cacctg, catgtg]	*RGS8*	85397
1	181828790	181828938	[catgtg]	*NCF2*	4688
1	200123540	200123592	-	*TMEM58*	149345
1	200123640	200123888	[cacctg]	*TMEM58*	149345
1	200830021	200830269	[cacctg]	N/A	N/A
1	201361984	201362132	[cacctg]	*ADORA1*	134
1	202390896	202391044	[caactg, catgtg]	*ETNK2*	55224
1	202714018	202714166	[caactg, catgtg]	N/A	N/A
1	218511926	218512074	[catttg]	*RAB3GAP2*	25782
1	222009349	222009497	[cacttg]	N/A	N/A
1	222870102	222870350	-	*CNIH3*	149111
1	225990520	225990668	[catgtg]	*C1orf142*	116841
1	234834062	234834310	-	*HEATR1*	55127
1	242881397	242881445	[catgtg]	*C1orf121*	51029
1	245646616	245646764	-	*CIAS1*	114548
1	246682971	246683119	-	*OR2T2*	401992
1	246856703	246856951	[catctg, cacctg, cagctg]	*OR2T11*	127077
1	246880459	246880607	[cacctg]	*OR2T27*	403239
1	247113752	247113900	[caactg]	N/A	N/A
1	247116752	247117000	[cacctg]	N/A	N/A
2	60836987	60837235	-	*PAPOLG*	64895
2	73315914	73316166	[catttg, cacttg]	*CCT7*	10574
2	74456838	74457186	[catttg, cacttg, catgtg]	*DCTN1*	1639
2	96059305	96059453	[cacttg, caactg]	*LOC150763*	150763
2	114073858	114074106	[cagctg]	*MGC13005*	84771
2	114102875	114103023	[catctg, caactg, catgtg]	*RABL2A*	11159
2	202354353	202354601	-	*ALS2*	57679
2	218518857	218519105	-	*TNS1*	7145
2	219600978	219601026	[cacctg, caactg]	*DKFZp434O0527*	255101
2	219745910	219746165	[cacctg, cagctg, catatg]	N/A	N/A
2	233028209	233028267	-	*ALPI*	248
2	233442538	233442686	-	*UNQ830*	389084

Supplementary Table 1. MYCN binding sites common to all NB cell lines

Chromosome	Start position	End position	E-boxes	Nearest Gene	Entrez Gene ID
3	8668588	8668936	[cacctg, catgtg]	*C3orf32*	51066
3	39122895	39122943	-	*GORASP1*	64689
3	42108050	42108198	[catgtg]	*TRAK1*	22906
3	42820362	42820610	-	*LOC645943*	645943
3	47459337	47459585	[cacttg, catgtg]	*SCAP*	22937
3	48238766	48238814	-	*CAMP*	820
3	51974343	51974591	[catctg, catgtg]	*PCBP4*	57060
3	52456934	52457182	[cacgtg, cagctg]	*SEMA3G*	56920
3	52784030	52784085	-	*ITIH1*	3697
3	55668821	55669081	-	*C3orf51*	711
3	55669121	55669279	[caactg]	*C3orf51*	711
3	62335852	62336001	-	*ZNF312*	55079
4	915162	915310	[cacgtg]	*GAK*	2580
4	8935342	8935391	-	*DUB3*	402164
4	66217352	66217700	-	*EPHA5*	2044
5	947012	947360	[cacctg, caactg, catgtg, cagctg]	*TRIP13*	9319
5	126142240	126142597	[catttg, cacttg, cacctg]	*LOC644760*	644760
5	131422746	131423094	[cacctg, catgtg]	*IL3*	3562
5	134899989	134900337	[catctg, cacctg, cagctg]	*NEUROG1*	4762
5	135195664	135195912	[catctg, cacctg]	N/A	N/A
5	135390779	135390827	[catctg]	*TGFBI*	7045
5	136864389	136864637	[cacttg, cacctg]	*SPOCK1*	6695
5	136864789	136864837	[cacttg]	*SPOCK1*	6695
5	139920976	139921224	-	*SRA1*	10011
5	140731246	140731494	[catctg, catgtg]	*PCDHGA6*	56109
5	140748289	140748637	[cacctg]	*PCDHGB4*	8641
5	141320412	141320560	[catctg]	*PCDH12*	51294
5	153805077	153805225	-	*SAP30L*	79685
5	176005165	176005413	[cacctg, catgtg]	*FLJ36951*	253314
5	176668920	176669168	[cagctg]	N/A	N/A
5	177587726	177587784	[catgtg]	N/A	N/A
6	148528	148576	-	*FLJ43763*	642316
6	3202393	3202541	[catctg]	*RP11-506K6.3*	389362
6	27970608	27970757	[caactg]	*HIST1H2AM*	8336
6	29663662	29663910	-	*OR2H2*	7932
6	29704178	29704426	-	*GABBR1*	2550
6	30050571	30050619	[cagctg]	*HCG9*	10255
6	30957440	30957588	-	*DDR1*	780
6	31008860	31008908	-	*SFTPG*	389376
6	31650407	31650455	-	*TNF*	7124
6	31759171	31759519	[cagctg]	*LY6G5C*	80741
6	31940917	31941365	[cacttg, catgtg]	*NEU1*	4758
6	32046031	32046279	-	*STK19*	8859
6	32058180	32058428	[catctg, cagctg]	*C4B*	721
6	32091017	32091165	[catctg, cagctg]	*C4A*	720
6	32126034	32126382	[cacttg, cacgtg]	*TNXB*	7148
6	32228379	32228527	-	*PRRT1*	80863
6	32267844	32268192	-	*GPSM3*	63940
6	32920045	32920393	[caactg]	*PSMB8*	5696
6	32928316	32928464	-	*PSMB9*	5698
6	33271360	33271608	[cacgtg]	*COL11A2*	1302
6	33364256	33364504	-	*WDR46*	9277
6	41823750	41824098	[catttg, catatg]	*PGC*	5225
6	43060717	43060965	[catctg]	*PPP2R5D*	5528
6	43089037	43089285	-	*MEA1*	4201
6	43295621	43295869	-	N/A	N/A
6	45495596	45495944	[cacctg]	*RUNX2*	860
6	75974041	75974189	[cagctg]	*COL12A1*	1303
6	112481875	112482123	-	*WISP3*	8838
6	160245363	160245514	[catttg, cacctg]	*MAS1*	4142
6	169935021	169935269	[cacctg, caactg]	*C6orf208*	80069
7	5436035	5436083	-	N/A	N/A
7	7646270	7646320	-	*RPA3*	6119
7	15692482	15692830	[catctg, catatg]	*MEOX2*	4223
7	27206872	27207033	[catatg]	*HOXA13*	3209
7	30978373	30978421	-	*GHRHR*	2692
7	45163324	45163572	-	*RAMP3*	10268
7	72134159	72134207	-	*LOC441257*	441257
7	93043337	93043585	-	*CALCR*	799
7	136678325	136678573	[cagctg]	*PTN*	5764
7	142286166	142286414	[cacctg, catgtg, catatg]	*TRPV6*	55503
7	142286466	142286516	-	*TRPV6*	55503
7	143378628	143378680	-	*OR2A5*	393046

Supplementary Table 1. MYCN binding sites common to all NB cell lines

Chromosome	Start position	End position	E-boxes	Nearest Gene	Entrez Gene ID
7	143620806	143621154	[catttg]	*ARHGEF5*	7984
7	149191265	149191513	[cacttg]	N/A	N/A
7	150382651	150382699	-	N/A	N/A
7	150607681	150607729	-	*SMARCD3*	6604
7	155021051	155021099	-	*LOC285888*	285888
8	30707436	30707584	[cacttg, catctg, caactg]	*GSR*	2936
8	38358072	38358121	-	*WHSC1L1*	54904
8	62363193	62363341	[cacctg]	*MGC34646*	157807
8	99507526	99507774	[catgtg]	*KCNS2*	3788
8	119192383	119192631	[catgtg, cagctg]	*EXT1*	2131
8	145133234	145133382	-	*PARP10*	84875
8	145155580	145155628	-	*SPATC1*	375686
9	16033	16181	[caactg]	*FLJ00038*	375690
9	26445	26793	[catttg, catctg, cacctg, caactg]	*RP11-34P13.4*	654412
9	23814029	23814377	[caactg]	*ELAVL2*	1993
9	34369414	34369462	-	*KIAA1161*	57462
9	34642632	34642780	[cagctg]	*IL11RA*	3590
9	35105426	35105674	-	*KIAA1539*	80256
9	45620955	45621303	[catttg, catctg, cacgtg]	*LOC504188*	504188
9	46280179	46280327	[cacgtg, caattg]	*LOC158318*	158318
9	123542085	123542333	[cacctg, cagctg]	*DAB2IP*	153090
9	130839347	130839695	[catttg, cacttg]	*FAM73B*	84895
10	3205519	3205567	[cacctg]	*PITRM1*	10531
10	43420661	43420910	[catgtg, cagctg]	*ZNF485*	220992
10	50484147	50484195	-	*CHAT*	1103
10	50484247	50484395	-	*CHAT*	1103
10	50916992	50917240	[cacctg]	*CTGLF1*	119016
10	102035790	102035938	-	*BLOC1S2*	282991
10	103579636	103580084	[cacctg, caactg]	*KCNIP2*	30819
10	103583336	103583584	[catctg, catgtg]	*KCNIP2*	30819
10	106387949	106388197	[catctg, cacctg, catgtg]	*SORCS3*	22986
10	123958847	123958995	[cacctg]	*TACC2*	10579
11	6237981	6238329	[cacctg]	*CCKBR*	887
11	8016056	8016104	-	*TUB*	7275
11	17694635	17694983	-	*MYOD1*	4654
11	57174077	57174325	-	*YPEL4*	219539
11	64169329	64169577	-	*NRXN2*	9379
11	65356393	65356641	-	*FLJ30934*	254122
11	66072667	66072915	[cacctg, catgtg]	*ZDHHC24*	254359
11	71175016	71175164	-	*FAM86C*	55199
11	74540111	74540259	[catgtg, cagctg]	*SLCO2B1*	11309
11	117551775	117552023	[catttg]	*SCN2B*	6327
12	14024570	14025018	[catctg, cagctg]	*GRIN2B*	2904
12	50731858	50732206	[caactg]	*NR4A1*	3164
12	53043976	53044024	-	*GPR84*	53831
12	54651227	54651577	[catttg, cacttg, cacctg]	*RAB5B*	5869
12	116285216	116285364	[caactg]	*NOS1*	4842
14	20580951	20580999	-	*RNASE7*	84659
14	23065805	23066153	[catctg, cacctg, caactg]	*ZFHX2*	85446
14	23117726	23117974	-	*JPH4*	84502
14	69616301	69616749	[cacttg, cacctg]	N/A	N/A
14	70137285	70137533	-	*MED6*	10001
14	74600031	74600279	[cagctg]	*ACYP1*	97
14	92187647	92187895	-	*RIN3*	79890
15	26696029	26696185	[catctg, catgtg]	*LOC440248*	440248
15	43195464	43195612	-	*DUOX2*	50506
15	60334243	60334591	[catctg, caactg]	N/A	N/A
16	28763913	28764161	[cacttg]	*TUFM*	7284
16	32171844	32172092	-	*TP53TG3*	24150
16	33112688	33112836	[cagctg]	*TP53TG3*	24150
16	49204347	49204595	[caactg]	N/A	N/A
16	65523756	65523804	-	*CES2*	8824
16	65983801	65983849	-	*CGI-38*	51673
16	68762929	68763177	[cacctg]	*MGC34761*	283971
17	3488922	3489170	[catctg, cagctg]	*CARKL*	23729
17	4813377	4813425	-	*SPAG7*	9552
17	7263567	7263619	-	*LOC374768*	374768
17	7322245	7322593	[caactg]	*LOC643664*	643664
17	7559904	7560152	-	*DNHD3*	146754
17	18002185	18002533	[cacttg, cacctg, caactg, cagctg]	*MYO15A*	51168
17	19254469	19254817	[catttg, catgtg, cagctg]	*ZNF179*	7732
17	23668277	23668525	[cacgtg]	*TMEM97*	27346
17	24432701	24433050	-	*TIAF1*	9220

Supplementary Table 1. MYCN binding sites common to all NB cell lines

Chromosome	Start position	End position	E-boxes	Nearest Gene	Entrez Gene ID
17	24529947	24530295	[cacttg, catctg, cacctg]	*MYO18A*	399687
17	29928481	29928629	[caattg]	*TMEM132E*	124842
17	35035905	35035953	[cacgtg]	*PPP1R1B*	84152
17	58871233	58871281	-	*CYB561*	1534
17	68674965	68675313	[cacttg, cacctg, caactg]	*SSTR2*	6752
17	69719391	69719639	[caactg]	*TTYH2*	94015
17	75810882	75811230	[cagctg]	*SGSH*	6448
19	2652914	2653362	[cagctg]	*GNG7*	2788
19	4407646	4407694	-	*UBXD1*	80700
19	7599872	7599920	-	*XAB2*	56949
19	8793952	8794200	[catttg, cacttg, catctg, cacctg]	*ZNF558*	148156
19	12996434	12996882	[cacttg]	*NFIX*	4784
19	13090809	13091057	[catctg, catgtg]	*BTBD14B*	112939
19	15059077	15059225	[catctg, cacctg]	*OR1I1*	126370
19	15699797	15699983	[caactg]	N/A	N/A
19	15779698	15779746	-	*OR10H1*	26539
19	19185762	19185910	[catgtg]	*CSPG3*	1463
19	40337001	40337349	[catttg, catctg]	*FXYD5*	53827
19	47040072	47040420	-	*LYPD4*	147719
19	47193037	47193485	[cacctg, caactg]	*ATP1A3*	478
19	47263669	47263917	[cacctg]	*ZNF574*	64763
19	47938433	47938481	-	*PSG3*	5671
19	47963727	47963775	[caattg]	*PSG8*	440533
19	48115680	48115928	[catctg, cacgtg]	*PSG6*	5675
19	48224425	48224473	-	*PSG11*	5680
19	48280433	48280681	[catctg, catgtg, catatg]	*PSG2*	5670
19	48384457	48384505	-	*PSG5*	5673
19	48403595	48403643	-	*PSG4*	5672
19	50460186	50460334	[cacctg, catgtg]	N/A	N/A
19	50913176	50913224	[cacctg]	*FBXO46*	23403
19	55127430	55127678	[catctg, cacctg]	*NUP62*	23636
19	55951048	55951196	-	N/A	N/A
19	55962133	55962281	-	*GPR32*	2854
19	56002961	56003209	[cacttg]	*MGC13170*	84798
19	56009949	56010197	[catgtg]	*MGC45922*	284365
19	56068828	56069076	[cacctg, cagctg]	*KLK2*	3817
19	56318777	56319025	[cacctg, catatg]	*SIGLEC9*	27180
19	56565765	56565813	-	*CLDND2*	125875
19	63410779	63411327	[cacctg, catgtg, cacgtg, cagctg]	*ZNF274*	10782
20	25792688	25792736	[cacttg]	N/A	N/A
20	30004523	30004871	[catgtg]	*PDRG1*	81572
20	30261157	30261405	[cacctg, caactg, catgtg]	*PLAGL2*	5326
20	39363210	39363266	-	*ZHX3*	23051
20	61359487	61359635	-	*C20orf58*	128414
21	29286546	29286894	-	*ZNF294*	26046
X	100765398	100765649	[catctg]	*LOC643785*	643785
