# Supplementary material for: Global MYCN Transcription Factor Binding Analysis in Neuroblastoma Reveals Association with Distinct E-Box Motifs and Regions of DNA Hypermethylation
Source: PLoS One. 2009 Dec 4;4(12):e8154. doi: 10.1371/journal.pone.0008154 (PMC2781550; doi:10.1371/journal.pone.0008154)
Supplement: Table S2 — Gene ontology of MYCN target genes. (0.07 MB PDF) [file pone.0008154.s008.pdf]

Supplementary Table 2. Gene Ontology of MYCN Target Genes

Cell line comparison	Term	Count	%	Bonferroni score
Unique Kelly Vs SKNAS (1709)	BP00044:mRNA transcription regulation	749	44.14	1.18E-17
	BP00286:Cell structure	310	18.27	7.33E-14
	MF00042:Nucleic acid binding	606	35.71	4.35E-12
	BP00143:Cation transport	358	21.1	1.27E-11
	MF00243:DNA helicase	171	10.08	1.56E-09
	BP00071:Proteolysis	404	23.81	1.64E-09
	BP00104:G-protein mediated signaling	321	18.92	2.24E-09
	BP00102:Signal transduction	336	19.8	1.45E-08
	BP00150:MHCI-mediated immunity	398	23.45	1.67E-07
	MF00213:Non-receptor serine/threonine protein kinase	342	20.15	4.33E-07
	BP00142:Ion transport	204	12.02	2.64E-06
	BP00149:T-cell mediated immunity	308	18.15	5.24E-06
	MF00242:RNA helicase	150	8.84	1.00E-05
	BP00103:Cell surface receptor mediated signal transduction	214	12.61	2.34E-05
	MF00101:Guanyl-nucleotide exchange factor	220	12.96	7.42E-05
	BP00111:Intracellular signaling cascade	101	5.95	1.54E-04
	BP00064:Protein phosphorylation	189	11.14	2.03E-04
	MF00131:Transferase	190	11.2	4.63E-04
	MF00108:Protein kinase	145	8.54	8.50E-04
	MF00072:Translation initiation factor	117	6.89	2.63E-03
	BP00026:Cholesterol metabolism	86	5.07	2.84E-03
	MF00034:Voltage-gated potassium channel	112	6.6	4.55E-03
	MF00262:Non-motor actin binding protein	269	15.85	6.19E-03
	BP00274:Cell communication	112	6.6	7.33E-03
	BP00282:Mitosis	86	5.07	9.53E-03
Unique SKNAS Vs Kelly (888)	BP00044:mRNA transcription regulation	367	41.7	1.10E-04
	BP00071:Proteolysis	207	23.52	5.18E-04
Unique SHEP Untreated Vs SHEP Treated (2688)	BP00044:mRNA transcription regulation	1158	43.29	1.41E-21
	BP00071:Proteolysis	665	24.86	1.82E-20
	MF00213:Non-receptor serine/threonine protein kinase	575	21.5	3.72E-18
	BP00143:Cation transport	539	20.15	4.88E-13
	BP00104:G-protein mediated signaling	496	18.54	1.15E-12
	BP00102:Signal transduction	518	19.36	3.69E-11
	MF00042:Nucleic acid binding	911	34.06	5.44E-11
	BP00141:Transport	217	8.11	2.59E-09
	MF00101:Guanyl-nucleotide exchange factor	354	13.23	4.55E-09
	BP00286:Cell structure	426	15.93	1.10E-08
	BP00274:Cell communication	190	7.1	7.89E-08
	MF00262:Non-motor actin binding protein	441	16.49	5.20E-07
	BP00103:Cell surface receptor mediated signal transduction	328	12.26	6.14E-07
	BP00142:Ion transport	304	11.36	9.18E-07
	BP00199:Neurogenesis	155	5.79	4.04E-06
	BP00064:Protein phosphorylation	288	10.77	1.92E-05
	MF00243:DNA helicase	225	8.41	3.22E-05
	MF00108:Protein kinase	221	8.26	6.67E-05
	MF00224:KRAB box transcription factor	660	24.67	8.93E-05
	MF00230:Actin binding motor protein	204	7.63	1.22E-04
	MF00131:Transferase	287	10.73	1.48E-04
	MF00137:Glycosyltransferase	175	6.54	1.62E-04
	MF00250:Serine protease inhibitor	258	9.64	2.99E-04
	BP00193:Developmental processes	248	9.27	3.04E-04
	BP00149:T-cell mediated immunity	448	16.75	4.68E-04
	BP00067:Protein glycosylation	219	8.19	6.10E-04
	MF00222:Zinc finger transcription factor	314	11.74	6.86E-04
Unique SHEP Treated Vs SHEP Untreated (714)	N/A	N/A	N/A	N/A
