# Supplementary material for: Global MYCN Transcription Factor Binding Analysis in Neuroblastoma Reveals Association with Distinct E-Box Motifs and Regions of DNA Hypermethylation
Source: PLoS One. 2009 Dec 4;4(12):e8154. doi: 10.1371/journal.pone.0008154 (PMC2781550; doi:10.1371/journal.pone.0008154)
Supplement: Table S3 — Expression of genes (fold change <0.5 and >1.5) which are methylated in SK-N-AS and not methylated in Kelly. (0.07 MB PDF) [file pone.0008154.s009.pdf]

Supplementary Table 3. Expression of genes (fold change <0.5 and > 1.5) which are methylated in SK-N-AS and not methylated in Kelly

ID	Gene	Expression Kelly	Expression SKNAS	Fold difference (Kelly/SK-N-AS)
NM_000076	*CDKN1C*	288.4966	82.6921	3.49
NM_000173	*GP1BA*	609.8355	96.6783	6.31
NM_000233	*LHCGR*	37.8063	21.4821	1.76
NM_000329	*RPE65*	108.0869	62.0755	1.74
NM_000492	*CFTR*	25.1296	135.6272	0.19
NM_000551	*VHL*	2302.923	847.4041	2.72
NM_000599	*IGFBP5*	624.1751	2926.6627	0.21
NM_000732	*CD3D*	96.7686	21.6534	4.47
NM_000953	*PTGDR*	150.407	36.7319	4.09
NM_000985	*RPL17*	867.0863	2797.0863	0.31
NM_001002755	*NFU1*	178.9334	562.4031	0.32
NM_001007225	*IGF2BP2*	198.0318	1578.1714	0.13
NM_001013732	*C6orf138*	180.2989	30.1061	5.99
NM_001024938	*SLC2A11*	880.202	200.4397	4.39
NM_001031679	*MSRB3*	118.7408	1129.8113	0.11
NM_001039132	*ICAM4*	85.8194	214.8113	0.40
NM_001977	*ENPEP*	15.9723	390.8041	0.04
NM_001990	*EYA3*	166.9619	547.1014	0.31
NM_002317	*LOX*	537.1413	299.7013	1.79
NM_002829	*PTPN3*	31.4976	761.7735	0.04
NM_002925	*RGS10*	1276.4247	413.6097	3.09
NM_003020	*SCG5*	1828.1655	256.7771	7.12
NM_003532	*HIST1H3E*	133.1519	560.3663	0.24
NM_003665	*FCN3*	119.9604	256.4425	0.47
NM_004155	*SERPINB9*	1326.2134	45.9489	28.86
NM_004289	*NFE2L3*	34.1333	13838.74	0.00
NM_004617	*TM4SF4*	379.0524	79.7111	4.76
NM_004650	*PNPLA4*	196.3686	79.5603	2.47
NM_004686	*MTMR7*	260.3096	40.1468	6.48
NM_004714	*DYRK1B*	526.5023	207.8094	2.53
NM_005270	*GLI2*	26.1366	578.4042	0.05
NM_005442	*EOMES*	70.4355	34.1639	2.06
NM_007017	*SOX30*	136.5242	90.3808	1.51
NM_007033	*RER1*	1030.5313	663.6963	1.55
NM_007345	*ZNF236*	1104.2563	2572.5363	0.43
NM_007366	*PLA2R1*	251.4983	127.5217	1.97
NM_012296	*GAB2*	2715.9238	535.3841	5.07
NM_012404	*ANP32D*	28.7409	67.4476	0.43
NM_014237	*ADAM18*	30.4261	14.9228	2.04
NM_014650	*ZNF432*	514.2578	280.6332	1.83
NM_014926	*SLITRK3*	416.2912	97.5566	4.27
NM_015052	*HECW1*	228.6122	32.2758	7.08
NM_015490	*SEC31B*	24.4782	98.5112	0.25
NM_015559	*SETBP1*	154.4634	387.5539	0.40
NM_015567	*SLITRK5*	3829.0363	2506.2563	1.53
NM_015873	*VILL*	204.4507	34.6714	5.90
NM_017842	*FLJ20489*	179.5895	382.0225	0.47
NM_017898	*MOSC2*	307.9111	105.649	2.91
NM_018216	*PANK4*	2201.0236	568.481	3.87
NM_018238	*AGK*	1906.9813	1193.4213	1.60
NM_018383	*WDR33*	514.5395	308.2894	1.67
NM_018724	*IL20*	33.1707	215.0863	0.15
NM_018891	*LAMC2*	145.4018	57.6364	2.52
NM_019851	*FGF20*	165.2332	333.4117	0.50
NM_020299	*AKR1B10*	84.8377	48.6713	1.74
NM_020436	*SALL4*	1150.3663	73.8014	15.59
NM_020731	*AHRR*	98.2403	382.2563	0.26
NM_020989	*CRYGC*	426.9263	30.0554	14.20
NM_022134	*GAL3ST2*	317.8663	26.3312	12.07
NM_022571	*GPR135*	135.4898	352.1463	0.38
NM_024667	*VPS37B*	495.3946	1579.5536	0.31
NM_024848	*MORN1*	419.8113	268.0913	1.57
NM_024885	*TAF7L*	31.4252	93.3321	0.34
NM_030933	*C1orf14*	132.5015	44.8116	2.96
NM_031283	*TCF7L1*	14.9331	239.543	0.06
NM_031291	*SLC25A31*	15.7247	62.6676	0.25
NM_031917	*ANGPTL6*	84.5307	48.957	1.73
NM_032047	*B3GNT5*	523.4263	1853.7013	0.28
NM_032265	*ZMYND15*	30.3948	143.4867	0.21
NM_032515	*BOK*	339.2013	150.7031	2.25
NM_033035	*TSLP*	313.0303	143.1483	2.19
NM_033184	*KRTAP2-4*	95.5134	60.5758	1.58
NM_033195	*LDHAL6B*	247.3713	15.0724	16.41
NM_033210	*ZNF502*	207.0836	102.3925	2.02
NM_033515	*ARHGAP18*	189.981	3514.9712	0.05
NM_052839	*PANX2*	282.4813	158.0921	1.79
NM_052858	*MARVELD3*	455.1939	210.1484	2.17
NM_052971	*LEAP2*	91.8631	43.6186	2.11
NM_080431	*ACTRT2*	252.098	30.7319	8.20
NM_080662	*PEX11G*	35.1353	90.8669	0.39
NM_138428	*C1orf212*	190.3425	475.6786	0.40

Supplementary Table 3. **Expression of genes (fold change <0.5 and > 1.5) which are methylated in SK-N-AS and not methylated in Kelly**

ID	Gene	Expression Kelly	Expression SKNAS	Fold difference (Kelly/SK-N-AS)
NM_139171	*STARD6*	258.8025	22.7867	11.36
NM_144668	*WDR66*	529.7563	2704.8113	0.20
NM_144715	*EFHB*	184.2125	112.8954	1.63
NM_145653	*TCEB3C*	51.0697	122.3428	0.42
NM_145792	*MGST1*	381.1515	22.8913	16.65
NM_147168	*C9orf24*	88.2078	23.3119	3.78
NM_152393	*KBTBD5*	522.2563	62.4392	8.36
NM_152554	*C6orf195*	306.1517	85.8158	3.57
NM_152721	*DOK6*	277.8432	1157.7981	0.24
NM_153339	*PUSL1*	963.5363	282.0913	3.42
NM_153347	*TMEM86A*	13.6159	67.1676	0.20
NM_172241	*CTAGE1*	171.7106	52.6326	3.26
NM_173488	*SLCO6A1*	93.9162	43.6701	2.15
NM_176096	*CDK5RAP3*	3094.4213	1336.4813	2.32
NM_178013	*PRIMA1*	1666.4213	119.3492	13.96
NM_178172	*LOC338328*	199.5503	99.2688	2.01
NM_178865	*SERINC2*	57.2732	27.2869	2.10
NM_181709	*FAM101A*	206.5957	828.5378	0.25
NM_181756	*ZNF233*	406.0996	209.4456	1.94
NM_182589	*HTR3E*	31.2346	67.5695	0.46
NM_198274	*SMYD1*	45.2863	166.1499	0.27
NM_203407	*LOC340602*	437.6631	62.9418	6.95
NM_203411	*TMEM88*	153.3727	38.5892	3.97
