# Supplementary material for: Global MYCN Transcription Factor Binding Analysis in Neuroblastoma Reveals Association with Distinct E-Box Motifs and Regions of DNA Hypermethylation
Source: PLoS One. 2009 Dec 4;4(12):e8154. doi: 10.1371/journal.pone.0008154 (PMC2781550; doi:10.1371/journal.pone.0008154)
Supplement: Table S4 — Expression of genes (fold change <0.5 and >1.5) which are methylated in Kelly and not methylated in SK-N-AS. (0.07 MB PDF) [file pone.0008154.s010.pdf]

Supplementary Table 4. Expression of genes (fold change <0.5 and > 1.5) which are methylated in Kelly and not methylated in SK-N-AS

ID	Gene	Expression Kelly	Expression SKNAS	Fold change (SK-N-AS / Kelly)
NM_000217	*KCNA1*	39.789	91.3314	2.30
NM_000332	*ATXN1*	326.7013	669.8113	2.05
NM_000612	*IGF2*	14743.0363	319.9213	0.02
NM_000727	*CACNG1*	67.0349	188.8402	2.82
NM_000800	*FGF1*	18.5896	94.7458	5.10
NM_000960	*PTGIR*	126.5368	312.7987	2.47
NM_001040084	*ANXA8*	57.2426	149.7443	2.62
NM_001089	*ABCA3*	1770.8131	670.3292	0.38
NM_001222	*CAMK2G*	466.8745	1425.8863	3.05
NM_001382	*DPAGT1*	346.7563	903.5913	2.61
NM_001668	*ARNT*	152.5378	312.3663	2.05
NM_001887	*CRYBB1*	323.4763	77.287	0.24
NM_001939	*DRP2*	58.3419	357.1463	6.12
NM_002141	*HOXA4*	398.5313	132.268	0.33
NM_002152	*HRC*	180.536	16.364	0.09
NM_002236	*KCNF1*	160.202	54.1049	0.34
NM_002403	*MFAP2*	1564.0394	2407.9837	1.54
NM_002409	*MGAT3*	137.4284	209.5913	1.53
NM_002509	*NKX2-2*	112.5365	304.977	2.71
NM_002518	*NPAS2*	47.472	567.3742	11.95
NM_002763	*PROX1*	1146.4263	222.9213	0.19
NM_002870	*RAB13*	1358.0363	3778.1963	2.78
NM_002961	*S100A4*	144.2599	2049.1463	14.20
NM_002971	*SATB1*	1801.8128	493.4183	0.27
NM_003083	*SNAPC2*	282.3537	601.0766	2.13
NM_003407	*ZFP36*	317.148	1059.1204	3.34
NM_003604	*IRS4*	130.9885	58.2963	0.45
NM_003741	*CHRD*	154.7575	41.7692	0.27
NM_003897	*IER3*	739.9263	13237.4763	17.89
NM_004261	*SEPT15*	4084.9213	6407.4263	1.57
NM_004312	*ARR3*	48.049	150.8131	3.14
NM_004313	*ARRB2*	882.8352	231.5863	0.26
NM_004679	*VCY*	272.9813	64.0255	0.23
NM_004917	*KLK4*	898.0471	168.9665	0.19
NM_004923	*MTL5*	171.2565	41.2676	0.24
NM_004952	*EFNA3*	601.4763	94.0914	0.16
NM_004988	*MAGEA1*	1614.2284	22.8989	0.01
NM_004995	*MMP14*	324.9813	814.3163	2.51
NM_005328	*HAS2*	331.7013	2852.4213	8.60
NM_005428	*VAV1*	453.5913	140.4816	0.31
NM_005596	*NFIB*	105.7572	1179.5363	11.15
NM_005840	*SPRY3*	138.043	14.7545	0.11
NM_006187	*OAS3*	194.3113	1625.2563	8.36
NM_006317	*BASP1*	10616.4763	4721.0863	0.44
NM_006462	*RBCK1*	734.8163	1430.7563	1.95
NM_006531	*IFT88*	345.9763	1277.6413	3.69
NM_006772	*SYNGAP1*	112.7538	20.9968	0.19
NM_007068	*DMC1*	98.9882	35.9684	0.36
NM_012144	*DNAI1*	84.2273	15.1934	0.18
NM_012324	*MAPK8IP2*	1387.2563	155.4274	0.11
NM_012391	*SPDEF*	76.9287	170.7072	2.22
NM_014011	*SOCS5*	411.707	1161.4319	2.82
NM_014023	*WDR37*	125.8726	203.4654	1.62
NM_014078	*MRPL13*	4210.2744	6954.7805	1.65
NM_014589	*PLA2G2E*	45.1406	68.6254	1.52
NM_015087	*SPG20*	146.0989	586.5975	4.02
NM_015944	*AMDHD2*	644.2299	165.938	0.26
NM_016941	*DLL3*	494.0363	149.2035	0.30
NM_017521	*FEV*	2703.2272	70.2985	0.03
NM_017596	*KIF21B*	1342.5385	383.3307	0.29
NM_017609	*C10orf92*	59.4093	132.659	2.23
NM_018056	*TMEM39B*	371.7209	888.5059	2.39
NM_018354	*C20orf46*	645.9076	194.3647	0.30
NM_020196	*XAB2*	336.2563	756.7563	2.25
NM_020742	*NLGN4X*	1515.1463	22.3044	0.01
NM_020777	*SORCS2*	45.8966	168.9547	3.68
NM_021035	*ZNFX1*	251.1413	1021.4213	4.07
NM_021185	*C19orf15*	228.5684	427.3526	1.87
NM_021192	*HOXD11*	44.4997	407.6463	9.16
NM_021193	*HOXD12*	161.2459	77.2241	0.48
NM_022045	*MTBP*	201.3663	840.1413	4.17
NM_022046	*KLK14*	142.3525	287.2203	2.02
NM_022340	*ZFYVE20*	2041.2507	767.1444	0.38
NM_022661	*SPANXC*	55.8398	133.0891	2.38
NM_023068	*SIGLEC1*	167.2175	49.2688	0.29
NM_024017	*HOXB9*	115.0764	192.7185	1.67
NM_024526	*EPS8L3*	123.6178	383.4288	3.10
NM_024545	*SAP130*	408.7328	1357.6089	3.32

Supplementary Table 4. Expression of genes (fold change <0.5 and > 1.5) which are methylated in Kelly and not methylated in SK-N-AS

ID	Gene	Expression Kelly	Expression SKNAS	Fold change (SK-N-AS / Kelly)
NM_024584	*CCDC121*	172.311	41.2154	0.24
NM_024681	*KCTD17*	458.5884	216.1983	0.47
NM_024684	*C11orf67*	1287.5626	2629.2742	2.04
NM_030792	*GDPD5*	516.2872	86.7135	0.17
NM_031463	*HSDL1*	1556.2448	613.0151	0.39
NM_031500	*PCDHA4*	78.9679	159.587	2.02
NM_032160	*DSEL*	71.7894	282.4813	3.93
NM_032461	*SPANXB1*	33.5744	67.4402	2.01
NM_032488	*CNFN*	180.9214	368.5913	2.04
NM_032531	*KIRREL3*	58.6811	104.6938	1.78
NM_133367	*PAQR8*	618.0913	103.2312	0.17
NM_138962	*MSI2*	501.0692	933.8211	1.86
NM_145057	*CDC42EP5*	139.7682	265.166	1.90
NM_145662	*SPANXA2*	29.5414	130.4532	4.42
NM_147161	*ACOT11*	68.0651	125.2789	1.84
NM_152268	*PARS2*	227.3163	413.5313	1.82
NM_152296	*ATP1A3*	2342.6562	31.2417	0.01
NM_152458	*ZNF785*	18.4636	38.0651	2.06
NM_152536	*FGD5*	110.0035	23.9693	0.22
NM_153216	*POU5F2*	247.1247	83.2362	0.34
NM_153240	*NPHP3*	62.1465	375.717	6.05
NM_153371	*LNX2*	573.9509	968.7052	1.69
NM_173535	*CLEC4F*	411.7563	134.4906	0.33
NM_175607	*CNTN4*	505.7289	829.3853	1.64
NM_177998	*OTOP1*	44.1741	207.9778	4.71
NM_178275	*DKFZp434B1231*	139.814	48.8366	0.35
NM_178835	*LOC152485*	473.9763	1785.1463	3.77
NM_182533	*C1orf86*	93.5552	1152.7562	12.32
NM_194293	*XIRP1*	318.0223	91.6166	0.29
NM_198188	*ASTN2*	1143.4105	336.4861	0.29
NM_198545	*C1orf187*	19.8314	94.1924	4.75
NM_198690	*KRTAP10-9*	21.3291	306.8702	14.39
NM_198699	*KRTAP10-12*	17.7213	177.9417	10.04
NM_199051	*FAM5C*	202.31	2243.9217	11.09
NM_207313	*TMEM132E*	129.0112	60.6661	0.47
NM_207336	*ZNF467*	34.3174	401.3663	11.70
