# Supplementary material for: Global MYCN Transcription Factor Binding Analysis in Neuroblastoma Reveals Association with Distinct E-Box Motifs and Regions of DNA Hypermethylation
Source: PLoS One. 2009 Dec 4;4(12):e8154. doi: 10.1371/journal.pone.0008154 (PMC2781550; doi:10.1371/journal.pone.0008154)
Supplement: Table S5 — Expression of genes (fold change <0.5 and >1.5) which are methylated and MYCN bound in Kelly and not methylated or MYCN bound in SK-N-AS. (0.04 MB PDF) [file pone.0008154.s011.pdf]

Supplementary Table 5. Expression of genes (fold change <0.5 and > 1.5) which are methylated and MYCN bound in Kelly and not methylated or MYCN bound in SK-N-AS

	Kelly (methylated; MYCN bound)	SK-N-AS (Not methylated; Not MYCN bound)	
**Genes**	**Expression**	**Expression**	**Fold difference (Kelly/SK-N-AS)**
ADAMTS5	171.4653	52.3432	3.28
CAMTA1	1446.8082	140.1791	10.32
CD4	409.8113	162.0918	2.53
CYHR1	726.5313	454.3663	1.60
EGFL8	543.9235	289.1715	1.88
KIFC2	47.063	244.7991	0.19
KIRREL	102.8582	436.7502	0.24
KLHL21	1395.0863	788.0863	1.77
MARK2	374.2013	792.8113	0.47
MRPL41	2652.3727	1595.5683	1.66
NME2	131.6008	61.0664	2.16
PNPLA7	292.6463	185.3113	1.58
SLC12A7	850.5231	167.9538	5.06
SOX2	513.2483	167.5893	3.06
SVOPL	78.3501	31.9713	2.45
TIGD3	36.3626	17.2809	2.10
