# Supplementary material for: Global MYCN Transcription Factor Binding Analysis in Neuroblastoma Reveals Association with Distinct E-Box Motifs and Regions of DNA Hypermethylation
Source: PLoS One. 2009 Dec 4;4(12):e8154. doi: 10.1371/journal.pone.0008154 (PMC2781550; doi:10.1371/journal.pone.0008154)
Supplement: Table S6 — Expression of genes (fold change <0.5 and >1.5) which are methylated and MYCN bound in Kelly and methylated but not MYCN bound in SK-N-AS. (0.03 MB PDF) [file pone.0008154.s012.pdf]

Supplementary Table 6. Expression of genes (fold change <0.5 and > 1.5) which are methylated and MYCN bound in Kelly and methylated but not MYCN bound in SK-N-AS

	Methylated; MYCN bound - KELLY	Methylated; not MYCN bound - SK-N-AS	
**Genes**	**Expression**	**Expression**	**Fold difference (Kelly/SK-N-AS)**
APOC2	170.9265	29.0364	5.886628508
ARMC3	191.9908	47.9211	4.006393843
DERL3	225.0285	100.0377	2.249436962
FASTK	720.8663	476.1463	1.51395968
GAST	247.9307	30.6855	8.079734728
GGTL4	210.8113	115.478	1.825553785
HAPLN1	54.2536	236.1308	0.229760794
HCST	106.3218	412.6487	0.257656937
LOC198437	238.4633	156.6434	1.522332253
LOC389458	170.2143	106.7043	1.595196257
RASIP1	69.1806	27.4443	2.520763874
Rgr	238.7628	46.346	5.151745566
SLC43A3	743.7783	145.8545	5.099453908
TA-NFKBH	397.906	160.6621	2.476663756
THRA	195.1957	123.3542	1.582400113
UBL4B	85.0352	56.4219	1.507131096
WDR86	32.3678	15.6785	2.064470453
