# Supplementary material for: Global MYCN Transcription Factor Binding Analysis in Neuroblastoma Reveals Association with Distinct E-Box Motifs and Regions of DNA Hypermethylation
Source: PLoS One. 2009 Dec 4;4(12):e8154. doi: 10.1371/journal.pone.0008154 (PMC2781550; doi:10.1371/journal.pone.0008154)
Supplement: Table S7 — Bi-sulphite sequencing of selected loci. (0.01 MB PDF) [file pone.0008154.s013.pdf]

Supplementary Table 7. **Bi-sulphite sequencing of selected loci**

			Bisulphite Sequencing †		MeDIP array analysis	
**Chromosomal Coordinates**	**Forward primer**	**Reverse primer**	**Kelly**	**SK-N-AS**	**Kelly**	**SK-N-AS**
chr2:15977981-15979025	TGTTTGTTGTTGAATGTGATTT	TAACCTTTACCTTCCCAAAAA	5/7	5/7	Methylated	Unmethylated
chr2:16023085-16023871	GAATTTTGGGGTTTTAGAGTGG	AAACAAATTCTCCCTCTTCACC	11/11	1/11	Methylated	Unmethylated
chr2:219565124-219566124	TTTTTTTTGTTAAGTTAAGTGTAGAGT	TCAACCCCCTATCCTATAAA	4/4	4/4	Methylated	Methylated
chr7:129924786-129926009	TTGTGTATGTGGGTTTTTTTTT	AACCCTTCTAAATTACACCCAC	9/10	10/10	Methylated	Methylated
chr7:130248215-130249462	AATTGTGGGGAGGAAAGTTAT	ATACTCCAACCAACCTCAAATT	7/7	0/7	Methylated	Unmethylated
chr8:41673063-41675565	TGAGTTTTTGGGGTTTGATTAG	TAATAAAAACTCCCTCATCCCC	21/21	18/21	Methylated	Methylated

† The denominator in each fraction represents the total number of CpG sites in the sequence and the numerator represents the methyalted CpG sites taken into account common methylated sites for both forward and reverse primers used for seqeuncing.
