# Supplementary material for: Global MYCN Transcription Factor Binding Analysis in Neuroblastoma Reveals Association with Distinct E-Box Motifs and Regions of DNA Hypermethylation
Source: PLoS One. 2009 Dec 4;4(12):e8154. doi: 10.1371/journal.pone.0008154 (PMC2781550; doi:10.1371/journal.pone.0008154)
Supplement: Table S8 — Taqman q-PCR primer and probes. (0.06 MB PDF) [file pone.0008154.s014.pdf]

Supplementary Table 8. Taqman q-PCR primers and probes

Entrez gene ID	Gene	Forward Primer	Reverse Primer	Probe
4601	*MXI1*	CTGGGAAAGCTCTGAGAATTAGGA	CTCTTTGCCCTGTGATCTTTGTACT	CAGACTTGGCAAACAC
6604	*SMARCD3*	GAGGCTGAGAGCTTCTGAATGG	GGTCCCAGGAAGCCTTTCAAG	CTGCACCAAAACCCA
3005	*H1F0*	TGATCGTCGTGTCATTGGATTCATC	GAGAGCCCAGAACCATGTCA	CCCGCCCCAGATCC
6790	*AURKA*	TCTGTGTTCTAGCCTTTCCAACTG	GTGAGCACACGAGGACAAGAA	CAGGACCGGATACATC
5878	*RAB5C*	CAGGGTGTTGGGTGGTAGAG	GCCTGGACTGCTCCAAAAC	TCGCCCAGGTCTCCAG
84515	*MCM8*	CAGAAAGACCCGAGGAGACAAC	GCCAGGGTGCCATTCTTG	CCCATTCCCCGCCCTC
4831	*NME2*	TGAGGTGCTGAGCAAAGCT	TCTGCTTGAGCCCTGGTTAAG	CTCTCCGCGCAAACC
3209	*HOXA13*	GAGTTGCGATTTTGCTCTTTCCA	CCGCAGGAACCGATAAGCA	CACCCTGCCACTGTTT
5757	*PTMA*	CCACACAACCGATTTCTTAGTTCGA	GGGCGATCCGAGAACAACA	ACGCTGGCCTTATCG
7869	*SEMA3B*	CCCAGCCTCTTTCCCCTAG	TGGAAGAGGAGTCAGCAGTGA	CCTCCCGCCCTCGCC
